# Very long intergenic non-coding (vlinc) RNAs directly regulate multiple genes in *cis* and *trans*

**DOI:** 10.1186/s12915-021-01044-x

**Published:** 2021-05-20

**Authors:** Huifen Cao, Dongyang Xu, Ye Cai, Xueer Han, Lu Tang, Fan Gao, Yao Qi, DingDing Cai, Huifang Wang, Maxim Ri, Denis Antonets, Yuri Vyatkin, Yue Chen, Xiang You, Fang Wang, Estelle Nicolas, Philipp Kapranov

**Affiliations:** 1 Institute of Genomics, School of Medicine, Huaqiao University, 668 Jimei Road, Xiamen, 361021 China; 2 AcademGene Ltd., 6, Acad. Lavrentjev ave, Novosibirsk, 630090 Russia; 3 SRC VB Vector Rospotrebnadzor, Novosibirsk, Koltsovo 630559 Russia; 4 School of Medicine, Xiamen University, Xiangan Southern Road, Xiamen, 361102 China; 5 LBCMCP, Centre de Biologie Intgrative (CBI), Universit de Toulouse, CNRS, UPS, Toulouse, France

**Keywords:** lncRNA, vlincRNA, Regulatory networks, RNA-chromatin interactions, Single-molecule sequencing, CRISPR/Cas13, RNA processing, Development, Cell cycle, Anti-cancer drugs

## Abstract

**Background:**

Themajority of the human genome is transcribed in the form of long non-coding (lnc) RNAs. While these transcripts have attracted considerable interest, their molecular mechanisms of function and biological significance remain controversial. One of the main reasons behind this lies inthe significant challenges posed by lncRNAs requiring thedevelopment of novel methods and concepts to unravel their functionality. Existing methods often lack cross-validation and independent confirmation by different methodologies and therefore leave significant ambiguity as to the authenticity of the outcomes. Nonetheless, despite all the caveats, it appears thatlncRNAs mayfunction, at least in part, by regulating other genes via chromatin interactions. Therefore, thefunction of a lncRNA could be inferred from the function of genes it regulates. In this work, we present a genome-wide functional annotation strategy for lncRNAs based on identification of their regulatory networks via theintegration of three distinct types of approaches: co-expression analysis, mapping of lncRNA-chromatin interactions, and assaying molecular effects of lncRNA knockdowns obtained using an inducible and highly specific CRISPR/Cas13 system.

**Results:**

We applied the strategy to annotate 407 very long intergenic non-coding (vlinc) RNAs belonging to a novel widespread subclass of lncRNAs. We show that vlincRNAs indeed appear to regulate multiple genes encoding proteins predominantly involved in RNA- and development-related functions, cell cycle, and cellular adhesion via a mechanism involving proximity between vlincRNAs and their targets in thenucleus. A typical vlincRNAs can be both apositive and negative regulator and regulate multiple genes both in *trans* and *cis*. Finally, we show vlincRNAs and their regulatory networks potentially represent novel components of DNA damage response and are functionally important for the ability of cancer cells to survive genotoxic stress.

**Conclusions:**

This study provides strong evidence for the regulatory role of the vlincRNA class of lncRNAs and a potentially important role played by these transcripts in the hidden layer of RNA-based regulation in complex biological systems.

**Supplementary Information:**

The online version contains supplementary material available at 10.1186/s12915-021-01044-x.

## Background

The lncRNAs embody a fascinating group of transcripts. Collectively, they represent the vast majority of the transcriptional output of the human genome in terms of the sequence complexity [1,3]. These transcripts have been implicated in virtually all major biological processes, including normal development and disease [4,9], and are hypothesized to represent a hidden layer of regulation in complex biological organisms [10, 11]. Still, relevance of this class of transcripts for most part remains controversial [12], with one of the main reasons behind it being lack of understanding of molecular mechanisms of function of these RNA species [13]. The progress of elucidating the functions and mechanisms of lncRNAs has been hampered by the challenges caused by the vastness of the genome covered by lncRNAs and unique features of these transcripts [13]. For example, in terms of relative mass, lncRNAs are enriched in the polyA RNA transcriptome and depleted in the fraction of the processed polyadenylated transcripts while the situation is opposite for protein-coding mRNAs [2]. In fact, these differences have led to a suggestion that lncRNAs do not possess sequence-specific functions, but might rather function via the process of transcription itself [14]. While novel approaches and strategies have been developed to address these challenges [15], they often lack cross-validation by independent methods and thus leave open the question about the amount of error and noise in the resulting lncRNA annotations [13].

Still, overall, the existing body of evidence for lncRNAs whose function has significant level of support points to the regulatory function of lncRNA, such as targeting components of chromatin modulation machinery to specific parts of the genome [4, 5]. Thus, identification of regulatory targets of lncRNAs likely holds the key to understanding their mechanisms of action. One popular approach to uncover regulatory networks of lncRNAs is based on co-expression strategy that assumes transcripts with similar expression patterns to also have functional relationships [16]. This concept has been widely applied to annotate functions of lncRNAs genome-wide in multiple systems [15]. However, the expression correlation in itself does not imply direct interactions and most of such networks have not been independently validated. Furthermore, the co-expression analysis has been performed in multiple biological systems using different gene expression analysis platforms, analytical methods, and sample types [13]. Therefore, the fraction of true signal in such studies is very hard to estimate without the validation and, in general, the guidelines for proper co-expression analysis do not exist yet to our knowledge.

These issues make it hard to ascertain the fraction of the true interacting partners in published co-expression networks, thus limiting their utility. On the other hand, more direct approaches, based either on (1) the affinity isolation and subsequent molecular analysis of complexes containing a lncRNA of interest or (2) on the analysis of RNA populations after knockdown or knockout of a specific lncRNA, potentially allow for more straightforward identification of its targets. However, to our knowledge, most of the networks identified in such experiments have not been independently validated either, thus leaving the questions of the technical noise levels in these networks as well as their biological significance open as well [13]. Furthermore, reverse genetics studies of lncRNAs have been hampered by a number of technical and interpretational issues associated with the existing techniques leading to significant ambiguity over the outcomes of these studies [12]. Thus, the lack of overall validation significantly limits the credibility of most lncRNA networks identified so far.

In this regard, it is important to note that the recent work by the FANTOM6 consortium has found correlation between the lncRNA networks and biological effects identified by targeting specific lncRNAs using antisense oligonucleotide (ASO) technology [17]. Moreover, the authors have found significant overlap among the networks found in different ASOs targeting the same target transcript supporting the validity of these findings [17]. However, the validation rate of these networks using a different technology (RNAi) was relatively low [17], possibly due to the different targeting modalities of ASOs and RNAi, thus leaving the question of cross-validation of the networks using a different technology open.

VlincRNAs belong to a recently identified subclass of lncRNAs. They are apparently un-spliced and polyA nuclear lncRNAs over 50kb and are quite common, with over 2000 vlincRNAs identified in the humans, transcribed from at least 10% of the human genome sequence [2, 18]. Since their recent discovery, vlincRNAs have been implicated in modulation of chromatin state in senescent cells [19] and control of replication timing of human chromosomes [20]. These transcripts provide an intriguing link between pluripotency and cancer [18]. Furthermore, vlincRNAs have been suggested to play a role in etiology or progression of acute lymphoblastic leukemia [21]. Recently, vlincRNAs were implicated in cellular response to anti-cancer drugs using CRISPR/Cas13 system [22]. However, the regulatory networks of most vlincRNAs remain unknown.

**Fig. 1:**
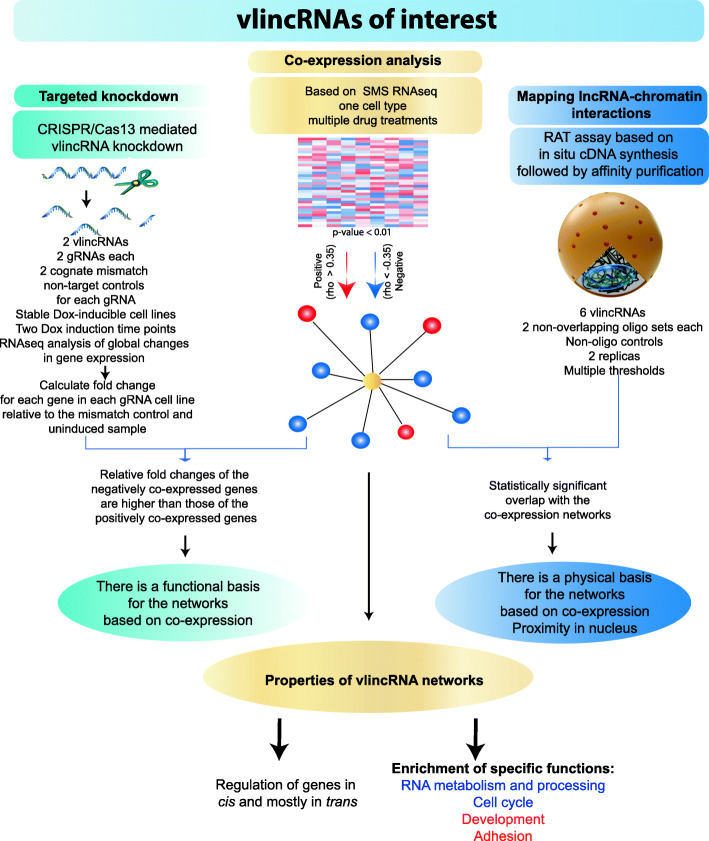
Flow chart diagram illustrating the overall concept of the project. The overall concept of the project, critical experimental and analytical steps and major conclusions are shown

The goal of this work is to identify lncRNA regulatory networks cross-validated by unrelated empirical approaches. We identify vlincRNA regulatory networks using a co-expression approach that theoretically should provide the most authentic networks of lncRNA regulatory targets and validate them using overlap with the maps of sites of vlincRNA-chromatin interactions as summarized in the Fig. 1. We further validate these networks by doing RNA-seq analysis of stable cell lines with inducible knockdown of the target vlincRNAs using the CRISPR/Cas13 system [23]. This system offers some crucial advantages over the existing reverse-genetics techniques, specifically it can directly target RNA thus providing unambiguousassignment of a phenotype to the target transcript and it allows for a closely matched control sequence for each guide (g) RNA [22, 23].

Based on the analysis of their regulatory networks, we show that each vlincRNA can regulate multiple genes located on the same (*cis*) or other chromosomes (*trans*). Furthermore, we found that the regulatory networks were quite reproducible under different treatment conditions. On one hand, vlincRNAs tend to negatively regulate multiple genes involved in RNA-related functions such as RNA transcription, metabolism, processing and splicing as well as cell cycle related functions. On the other hand, these lncRNAs appear to positively regulate genes involved in development, particularly nervous system development and adhesion. Finally, we show that expression analysis platform is critical for authenticity of the regulatory networks and careful consideration has to be given to the choice of RNA measurements and experimental design for such analysis.

## Results

### Co-expression strategy for lncRNA annotation

We hypothesized that a co-expression strategy based on a combination of the following features would likely generate the most accurate regulatory networks of lncRNAs (Fig. 1). First, we chose a 3rd generation next generation sequencing (NGS) single-molecule sequencing (SMS) platform as a foundation for estimation of expression of every lncRNA and mRNA species. This sequencing platform has relatively simple library preparation procedure that does not involve amplification steps [24] and thus more likely represents true original abundancies of various RNA species, especially in the low abundance range [25, 26]. The accuracy in this range is especially relevant for lncRNAs that tend to have low expression levels in general [3, 27]. Therefore, SMS could, in theory, provide a more accurate estimate of co-expression between lncRNAs and their potential target mRNAs. Second, we used a single cell type to generate the co-expression networks. While many publicly available expression datasets are available, reliance on them in the co-expression annotation of lncRNAs suffers from a major potential problem: lncRNAs are often expressed in just one or few cell types [3, 27]. Therefore, a co-expression analysis across multiple cell types would likely include many samples where any given lncRNA is either not expressed or expressed at noise levels and thus severely dilute the real correlation signals. Third, we used short time frames of transcriptome perturbing treatments (see below). We assumed that a co-expression analysis based on RNA levels measured shortly after the system is perturbed and forced to adapt by altering levels of various transcripts would more likely capture direct regulatory interactions as opposed to longer time treatments that could be diluted with indirect effects.

We have previously found expression of many vlincRNAs in a human leukemia cell line K562 [18]. This fact, together with the availability of multiple types of genomic data for this cell line from the ENCODE consortium [3], made K562 an attractive system for this study. The first step in our pipeline was to generate an expression database under multiple treatment conditions to calculate the co-expression of every vlincRNA with all protein-coding mRNAs. We profiled transcriptomes of K562 cell line after treatments with 29 inhibitors and anti-cancer drugs affecting diverse cellular pathways and functions (signaling pathways, cell cycle, DNA metabolism and repair, chromatin modifiers, etc.) (Additional file 1: Supplemental Table S1). As mentioned above, we used relatively short treatments of 3 and 6h for each drug.

**Fig. 2:**
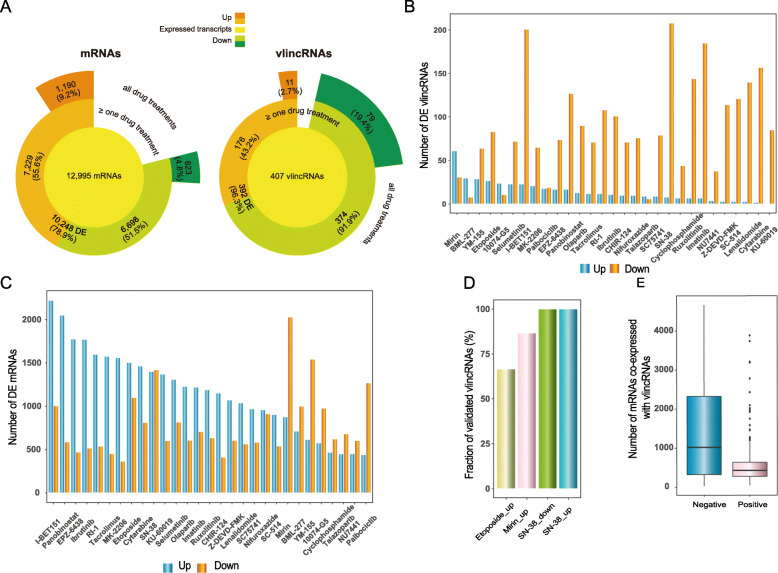
SMS-based expression and co-expression analyses for various drug treatments. **a** Distributions of the numbers of DE mRNAs (left) and vlincRNAs (right). The yellow inner circles represent mRNAs or vlincRNAs expressed in K562; the orange and green middle sections represent respectively up- or downregulated transcripts in at least one drug treatment; the orange and green outer sections represent respectively up- or downregulated transcripts in all drug treatments. **b**, **c** Numbers of DE vlincRNAs (**b**) and mRNAs (**c**) for each indicated treatment. The blue and orange bars represent respectively up- and downregulated transcripts. **d** Fractions of DE vlincRNAs validated by qPCR in each indicated treatment. **e** Box plots representing numbers genes found in either negative and positive co-expression-based vlincRNA networks

Total DNaseI-treated RNA from each sample was converted into cDNA using the not-so-random (NSR) hexamers devoid of sequences that bind to rRNAs [28] and analyzed using RNA-seq performed on the SMS platform. To estimate the degree of perturbation of the transcriptome by each drug, we estimated the number of differentially expressed (DE) up- or downregulated transcriptsboth protein-coding mRNAs and vlincRNAsdefined by fold change (FC) > 1.5 in both time points relative to the solvent (DMSO or water) controls for both 12,995 annotated genes expressed in K562 and 407 vlincRNAs detected previously in this cell line [18] (Fig. 2ac, Additional file 1: Supplemental Table S2). Overall, expression of 10,248 (78.9%) of the protein-coding genes changed under these conditions in at least one drug treatment with 7229 up- and 6698 downregulated genes (Fig. 2a, Additional file 1: Supplemental Table S2). The corresponding numbers for the vlincRNAs were 392 (96.3%) with 176 up- and 374 downregulated (Fig. 2a, Additional file 1: Supplemental Table S2). For any given drug treatment, we detected 1,190 (9.2%) and 623 (4.8%) up- or downregulated genes and correspondingly 11 (2.7%) and 79 (19.4%) vlincRNAs based on the corresponding median values across all treatment (Fig. 2a). Overall, vlincRNAs had a tendency to be downregulated as compared to known genes in response to drug treatments, suggesting potential negative correlation between these two types of transcripts (Fig. 2a, also see below).

The drugs varied significantly in respect to the effect on coding and non-coding transcriptomes (Fig. 2b, c, Additional file 1: Supplemental Table S2). Of the top three drugs that exhibited the largest upregulating effect on vlincRNAsmirin (inhibitor of MRE11, a component of the MRN complex), BML-277 (CHK2 inhibitor), and YM-155 (possible DNA intercalator) (Fig. 2b, Additional file 1: Supplemental Table S2)at least two are known to inhibit DNA damage sensing or response pathways (mirin and BML-277). DNA damage-related drugs also caused significant changes in the protein-coding transcriptome (Fig. 2c, Additional file 1: Supplemental Table S2). Still, the fraction of vlincRNAs upregulated in response to mirin and BML-277 treatments was higher than that of protein-coding mRNAs (Additional file 1: Supplemental Table S2). Furthermore, drugs that induced the highest fractions of expression of protein-coding genes affect epigenetic functions, such chromatin modifiers (panobinostat and EPZ-6438, inhibiting histone deacetylases and Ezh2 respectively) or readers of specific histone marks (bromodomain inhibitor I-BET151) and non-DNA damage related functions (Fig. 2c, Additional file 1: Supplemental Table S2). As such, it appears that the vlincRNA subclass of lncRNAs might be enriched in transcripts that participate in at least some cellular processes related to DNA damage.

To validate the reproducibility and authenticity of our expression analysis, we performed independent treatment experiments with three drugs (mirin, etoposide and SN-38) and analyzed the changes in expression of selected vlincRNAs in response to these drugs after 6h of the treatments using real-time PCR. We selected 42 differentially expressed (DE) vlincRNAs and, as expected, most (36, 85.7%) DE vlincRNAs could be validated (Fig. 2d, Additional file 1: Supplemental Table S3). Furthermore, of the 6 vlincRNAs that could not be validated in the real-time PCR experiments, 4 (66.7%) showed expected direction of the change albeit not reaching the FC of 1.5. As such, the DE analysis based on the SMS RNA-seq platform appears to capture authentic and reproducible expression changes.

We then generated a list of mRNAs co-expressed with each vlincRNA. The co-expression was defined as Spearman correlation of either >0.35 or < 0.35 between a vlincRNA and a protein-coding mRNA with the correlation significance *p* value <0.01 (Fig. 1) calculated on 64 samples (drug treatments and solvent-treated control samples). For each vlincRNA, we found between 134 and 5385 (median 1615) co-expressed transcripts using these thresholds. Interestingly, we have observed a much higher number of negatively correlated mRNAs than positively correlated ones with the medians of 430 and 1,022 for the positively and negatively co-expressed transcripts respectively, and the trend towards negative correlation was highly significant (*p* value <2.2E16, Wilcoxon signed rank test) (Fig. 2e, Additional file 1: Supplemental Table S4). Nonetheless, similar to the results reported earlier [29], genes positively correlating with vlincRNAs were enriched in the immediate vicinity of these transcripts. The median co-expression correlations between vlincRNAs and genes located within 5kb, 510kb, 10100kb, and > 100kb from each other were 0.44, 0.37, 0.38, and 0.38 respectively.

### Validation of the co-expression networks using lncRNA-chromatin interaction profiles

A major potential limitation of the co-expression strategy is that the expression correlation (positive or negative) can occur without direct physical or functional interactions between the correlated entities. Since a number of functionally characterized lncRNAs appear to regulate other genes by interacting and modulating their chromatin environment [4, 5], we assumed that the vlincRNAs could also function in the same fashion, as was in fact shown for the *VAD* vlincRNA [19]. Therefore, we validated the co-expression networks obtained in this study by mapping the sites of genome-wide RNA-chromatin interactions of selected vlincRNAs with the underlying key assumption that vlincRNAs should either interact or be in a relatively close proximity to their target genes (Fig. 1).

**Fig. 3:**
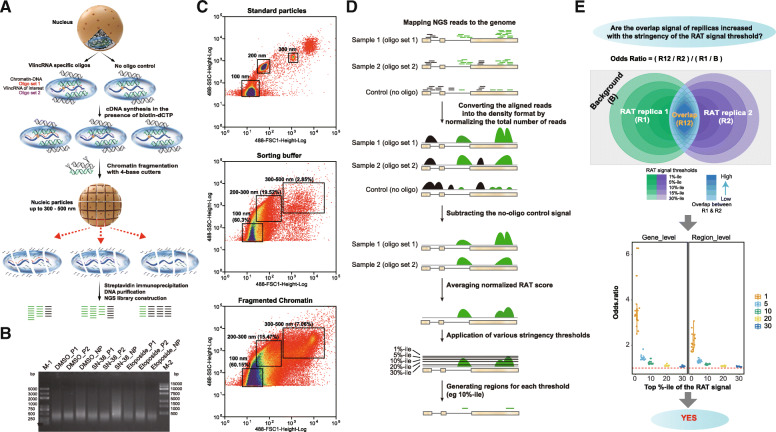
Description and validation of the RAT assay. **a** The flow diagram of the molecular biological part of the RAT assay. The light blue oval represents a region of the nucleus in the relative vicinity of a vlincRNA that would be co-purified with the vlincRNA by the RAT assay. The green and black lines represent DNA molecules that respectively are and are not located in the relative vicinity of vlincRNA. The red and purple lines represent specific oligonucleotides from the set 1 and 2 targeting each vlincRNA (short lines) and the cDNAs primed by these oligonucleotides (long lines). **b** An example of the DNA size distributions obtained after chromatin fragmentation in a typical RAT experiment for the DMSO- or drug-treated (etoposide or SN-38) samples. The assays performed with either the oligonucleotide set 1 (P1), 2 (P2), or the no-oligonucleotide control (NP) for the vlincRNA ID-1202. **c** Size distributions of particles obtained in a sorting experiment in the either the buffer (middle panel) and the buffer containing the chromatin fragmented using the conditions employed in a typical RAT experiment (bottom panel). The distribution of the particles with known sizes of 100, 200, and 300nm is shown in the top panel. Note the increase in the fraction of the particles in the 300500nm range in the fragmented chromatin sample vs the sorting buffer (7.06% vs 2.85%). **d** The flow diagram of the analytical part of the RAT assay. **e** Top: definition of the odds ratio and the depiction of the hypothesis tested in the part below. Bottom: box plots of the odds ratios of the overlaps between the two biological replicas of the RAT assay at the gene (left) and region (right) levels at different RAT signal thresholds (*X*-axes)

For this purpose, we adapted previously published RAT (reverse transcription-associated trap) approach [30, 31] that has two key advantages for the very long transcripts studied in this work (Fig. 3a). First, RAT relies on in situ reverse transcription inside crosslinked nuclei with oligonucleotides complementary to an RNA of interest and in presence of biotinylated dCTP to label RNA-chromatin complexes. Following the streptavidin immunoprecipitation, the bound chromatin regions are identified based on NGS analysis (Fig. 3a). The incorporation of biotin into the resulting cDNA obviates the need to design multiple closely spaced biotinylated oligonucleotide as in other techniques (e.g., ChIRP and similar methods) designed to map sites of interactions between a specific lncRNA and chromatin [32,34], which would be economically prohibitive for these very long transcripts. Second, chromatin fragmentation is conducted with restriction enzymes (Fig. 3a, b) that do not fragment RNA or single-stranded DNA unlike the other approaches that use sonication [32,34] that would likely break these very long transcripts.

Recently, a number of methods to detect genome-wide RNA-chromatin interactions were developed. However, one common feature of these methods (such as GRID-seq [35], MARGI [36], and Red-C [37]) was ligation of nearby DNA and RNA molecules using bridging oligonucleotides. The latter were in the range of ~4060 bases and could thus detect molecules separated by no more than 20nm given length of a nucleotide being 0.34nm. However, in our RATassays, the size of the chromatin particles after DNA fragmentation reached 300 to 500nm (Fig. 3c, Methods). Since all genomic regions would be expected to be located within such particles should be co-precipitated with the target transcript (Fig. 3a), this would mean that RAT is not limited to immediate interactions, but rather can measure much more distal proximity or colocalization between RNA and chromatin regions.

Since DNA damage-inducing drugs had the highest effect on the expression of vlincRNAs, we chose 6 vlincRNAs induced by the topoisomerase inhibitors (etoposide and/or SN-38) for the RAT analysis with an example of one such vlincRNA shown in Additional file 2: Supplemental Figure S1. The RAT procedure was performed on cells treated with either etoposide, SN-38 or DMSO. Overall, the RAT analysis was performed on 14 vlincRNA-treatment combinations with two biological replicas per combination with the goal of analyzing potential change in the networks in response to drug treatment. Each RAT assay was performed separately with 2 sets of non-overlapping oligonucleotides designed against the same vlincRNA (Fig. 3a, Methods). In addition, for each treatment, the RAT procedure was also performed without the oligonucleotides as a specificity control. Downstream analysis was performed using two levels of processed RAT signal: (1) average normalized RAT score calculated for every base pair in the human genome or (2) genomic region level obtained after application of thresholds of different stringency to the average normalized RAT score (Fig. 3d, Methods). The thresholds were defined based on the top 1 (most strict), 5, 10, 20, or 30 (least strict) percentile (%-ile) of the average normalized RAT score for each sample (Fig. 3d, Methods). Genes containing the RAT regions in their boundaries were considered co-localized with the corresponding vlincRNA.

As the first step in evaluation of the performance of the RAT approach, we estimated the overlap between RAT regions obtained from the biological replicas at either region or gene levels. In the former, exact genomic coordinates of the interacting regions had to be present in both replicas while in the latter genes had to contain interacting regions anywhere within their boundaries in both replicas but the coordinates of the interacting regions could be different. Overall, we found statistically significant overlap of the RAT signal between the replicas for every vlincRNA-treatment combination at both levels (Fig. 3e, Additional file 1: Supplemental Table S5). Furthermore, the overlaps of the RAT signal between the two replicas were statistically significant at multiple thresholds; however, as would be expected, the strengths of the overlaps, as measured by the odds ratios (defined in Fig. 3e) increased with the stringency of the RAT signal threshold (Fig. 3e, Additional file 1: Supplemental Table S5, Methods). In general, the odds ratios of the gene-level overlaps between the two replicas were consistently higher (Additional file 1: Supplemental Table S5). Therefore, unless specifically indicated, all analyses below were performed on genes containing the vlincRNA-chromatin interacting regions anywhere within their boundaries in both replicas (Methods).

**Fig. 4:**
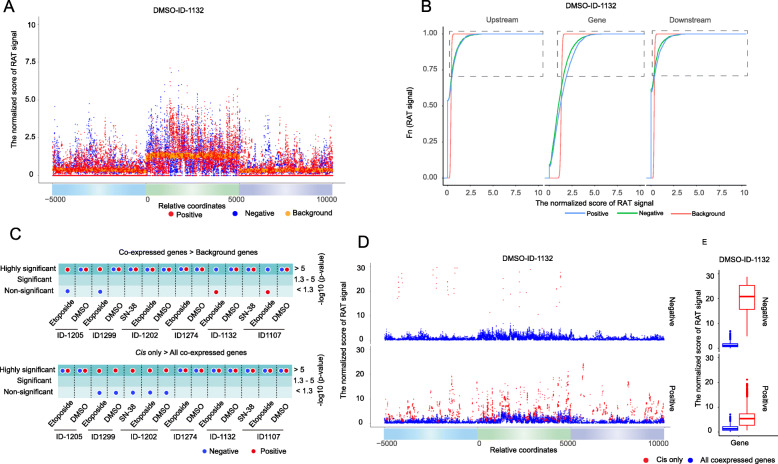
Patterns and statistical significance of enrichment of the RAT signal in the co-expressed genes. **a** Plots showing ANARS for gene bodies and 5kb flanking regions for all genes co-expressed with vlincRNA ID-1132 and the background genes. The sizes of the genic regions were scaled to 5kb. The ANARS shown in this example was calculated based on the RAT assay performed in the DMSO-treated cells. The ANARS for the positively, negatively and the control background genes is represented by respectively red, blue, and orange dots. **b** ECDF plots for the data shown in **a**. Note the shift to the right of the plots corresponding to the co-expressed genes signifying increase in the signal relative to the background genes. The top 30% of the data used for the statistical significance analysis are demarcated by the boxes. **c** Summary of the distribution of the statistical significance of enrichment of ANARS in the co-expressed vs the background genes (top) and *cis* vs all genes (bottom). **d** Plots showing ANARS for gene bodies and 5kb flanking regions for genes co-expressed with vlincRNA ID-1132 (and located on the same chromosome (*cis*, red dots) and all co-expressed genes (blue dots). The sizes of the genic regions were scaled to 5kb. **e** Boxplots of the data presented in **d** for positions with non-zero ANARS

To evaluate the relationship between co-expression and relative proximity in the nucleus between vlincRNAs and the co-expressed genes, for each vlincRNA, we measured average normalized aggregated RAT score (ANARS) in the boundaries of the corresponding co-expressed and background control genes and in their 5kb flanking regions (Additional file 3: Supplemental Figure S2, Methods). As shown in the Fig. 4a for one vlincRNA (ID-1132), the negatively and positively co-expressed genes had a tendency to have higher ANARS in gene bodies and their flanking regions than background genes. To formalize this observation, we generated empirical cumulative distribution function (ECDF) plots representing distribution of the ranked ANARS for the co-expressed and background genes (Fig. 4b, Additional file 4: Supplemental Figure S3, Methods). The ANARS of the co-expressed genes was consistently higher for the negative and positive co-expressed genes than the background genes, in gene bodies and in the flanking regions, for most vlincRNA-treatment conditions as shown in Fig. 4b for vlincRNA ID-1132 and in Additional file 4: Supplemental Figure S3 for all other vlincRNAs.

To test whether the difference is significant, we calculated *p* values of the enrichment of normalized RAT signal in the co-expressed relative to the background control genes. The statistical analysis was performed on the top 30% of the ranked ANARS values for the co-expressed and background genes, as illustrated by the regions of the ECDF plots demarcated by the boxes on Fig. 4b (Methods). The actual *p* values are given in the Additional file 1: Supplemental Table S6, and the results of the analysis are summarized in the Fig. 4c (gene bodies) and Additional file 5: Supplemental Figure S4 (gene bodies and flanking regions). Interestingly, the enrichment of the ANARS in the positively and negatively co-expressed genes compared to the background genes was statistically significant for most (12/14) vlincRNA-treatment combinations (Fig. 4c, Additional file 5: Supplemental Figure S4). Furthermore, the enrichment was statistically significant in all 14 combinations for either positively or negatively or both types of co-expressed genes (Fig. 4c, Additional file 5: Supplemental Figure S4).

Most co-expressed genes were located on chromosomes other than the one harboring thecorresponding vlincRNAs (*trans*). However, interestingly, the ANARS for the *cis* co-expressed genes (located on the same chromosome as the vlincRNA) had a tendency to be higher than that for all co-expressed genes as shown in the Fig. 4d, e for vlincRNA ID-1132 and in Additional file 6: Supplemental Figure S5 for all other vlincRNAs. We then estimated statistical significance of enrichment of the ANARS in the *cis* genes compared to all genes (Fig. 4c, Additional file 5: Supplemental Figure S4, Additional file 1: Supplemental Table S6). The enrichment was statistically significant among all samples for the positively co-expressed genes and for the majority (9/14) samples for the negatively co-expressed ones (Fig. 4c, Additional file 5: Supplemental Figure S4, Additional file 6: Supplemental Figure S5, Additional file 1: Supplemental Table S6). Taken together, these results provided a strong support that co-expressed genes were enriched using RAT procedure and therefore were located in the proximity of the corresponding vlincRNAs in the nucleus. However, positively co-expressed genes and those located on the same chromosome had consistently higher signal than the negatively co-expressed genes and those located on other chromosomes (see the Discussion section).

**Fig. 5:**
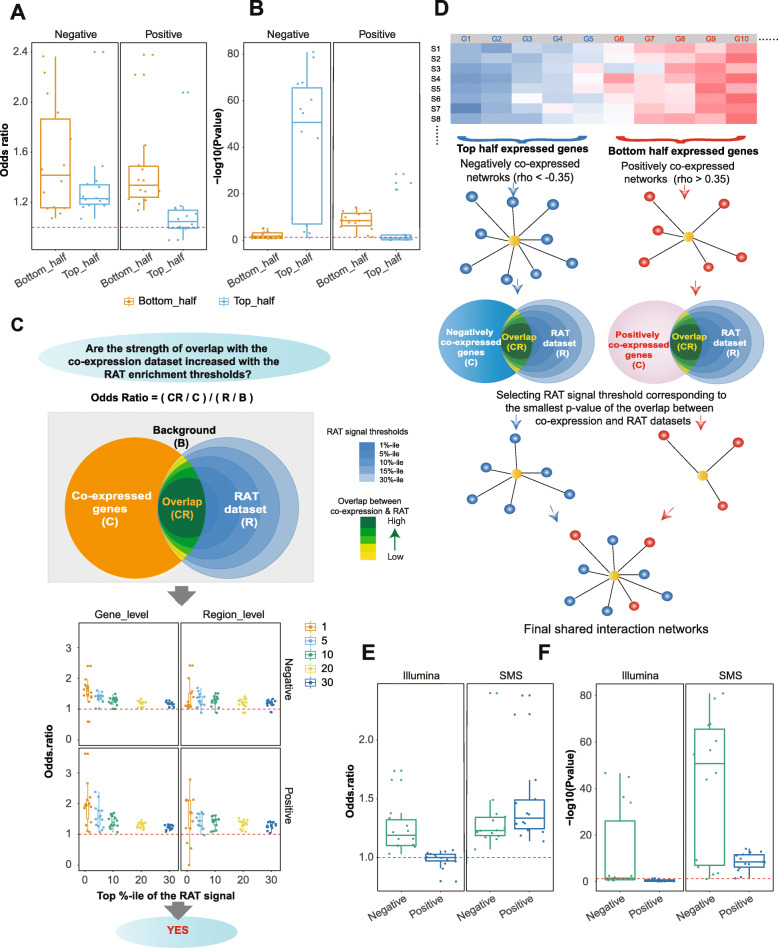
Validation of the co-expression derived networks using RAT assay. **a**, **b** Box plots of the odd ratios (**a**) and *p* values (**b**) of overlap between co-expression networks and the chromatin interaction datasets after stratifying the genes into the top and bottom half based on the expression. **c** Top: definition of the odds ratio and the depiction of the hypothesis tested in the part below. Bottom: box plots of the odds ratios of the overlaps between the co-expression networks and genes containing RAT regions at the gene (left) and region (right) levels at different RAT signal thresholds (*X*-axes). **d** A diagram illustrating selection of final RAT signal thresholds for each of the 14 vlincRNA-treatment combinations based on the best overlap with the co-expressed genes. **e**, **f** Overlap between the co-expression networks and genes containing RAT signals at the final RAT signal thresholds for the SMS and Illumina platforms. Odds ratios (**e**) and *p* values (**f**) are shown

We then estimated overlap between the co-expression dataset and genes containing RAT regions for every vlincRNA and made the following two observations. First, the significance of theoverlap depended on expression levels. Specifically, the low abundant genes had a much higher probability of having significant overlap between positively co-expressed genes and the genes showing evidence of co-localization compared to the highly abundant ones (Fig. 5a, b, Additional file 1: Supplemental Table S7). However, the trend was reversed for the genes negatively correlating with vlincRNAs (Fig. 5a, b, Additional file 1: Supplemental Table S7). We observed this trend for every vlincRNA and every treatment (Additional file 1: Supplemental Table S7). Therefore, to increase the signal to noise ratio, we first sorted genes by maximum expression among all samples and then filtered the negatively co-expressed genes by being in the top half of expressed genes and the positively co-expressed genes by being in the bottom half. Second, the strength of the overlap increased with the stringency of the RAT signal threshold as judged by the increasing odds ratios as illustrated in the Fig. 5c (Additional file 1: Supplemental Table S8). This result indicated that the RAT signal thresholds were indeed informative in enriching for co-localized vlincRNAs and their regulatory targets.

As the next step, we set to choose single RAT signal threshold individually for each of the 14 vlincRNA-treatment combinations based on the best overlap with the co-expressed genes as illustrated on Fig. 5d (Methods). Using these criteria, we found that a vlincRNA can be in the vicinity of 202030 (median 1104) and 47239 (median 123) negatively and positively co-expressed genes correspondingly. The odds ratios and the *p* values for the overlap between the final chromatin interaction maps and the negatively co-expressed genes ranged respectively from 1.07 to 2.4 (median 1.23) and from 1.16E81 to 7.82E2 (median 9.36E48) (Fig. 5e, f, boxplots marked SMS and Additional file 1: Supplemental Table S9). The corresponding values for the positively co-expressed genes were 1.14 to 2.38 (median 1.33) and 7.83E15 to 3.89E2 (median 3.91E9) (Fig. 5e, f, boxplots marked SMS and Additional file 1: Supplemental Table S10). The important outcome of this analysis was that majority of genes co-expressed with a vlincRNA (74.2% positive- or 81.7% negative-correlating transcripts) had evidence of co-localization with that vlincRNA.

### VlincRNAs directly regulate expression of genes in their regulatory networks

As the next step, to provide direct support for the regulatory effect of vlincRNAs, we assessed the effects of direct knockdown of 2 vlincRNAs achieved using the CRISPR/Cas13 system [23] on expression of genes in their regulatory networks (Fig. 1). We took advantage of the K562 cell line expressing doxycycline (Dox) inducible Cas13 that has been previously used by us to show biological relevance of vlincRNAs in a high-throughput screening [22]. In that study, a mixed population of cells with each cell stably expressing one of 588 individual gRNAs was subjected to a survival challenge with different anti-cancer drugs [22]. Here, we generated 8 stable cell lines expressing individual gRNAs found to make cells sensitive to genotoxic stress in that high-throughput screen and targeting 2 vlincRNAs [22]. For each vlincRNA, we generated 4 stable cell lines constitutively expressing 2 different targeting gRNAs and 2 cognate mis-match control gRNAs containing mutations in bases 1214 of the 28-mer gRNA as previously reported [22]. These mutations would abrogate the activity of the gRNA [23]. To avoid clonal effects, each cell line was represented by a mixed population of cells with different sites of lentivirus insertion.

**Fig. 6:**
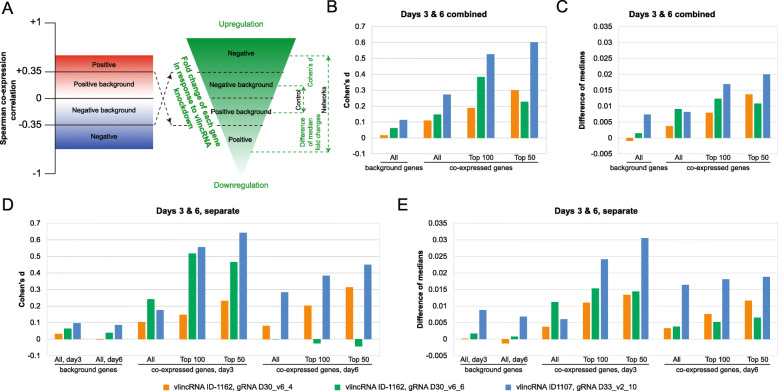
Effect of vlincRNA knockdowns using CRISPR/Cas13 on relative fold changes of the co-expressed genes. **a** Schematic representation of the expected connection between the either positively or negatively co-expressed genes (left) and the corresponding change in expression level in response to a vlincRNA knockdown (right). **be** Relative differences in the fold changes between negatively and positively co-expressed genes for each gRNA targeting-control pair (bottom). The relative differences were calculated as Cohens *d* metrics (**b**, **d**) or differences of medians (**c**, **e**) by either combining the data for both time points (3 and 6days) (**b**, **c**) or analyzing them separately (**d**, **e**). More details in the text

Each of the 8 cell lines was treated with Dox for 0, 3, or 6days, and the RNA population from each sample was subjected to RNA-seq analysis. Overall, we observed consistent knockdown in 3 out of 4 gRNAs with an average depletion of 20.4% compared to day 0 and the non-targeting control gRNAs based on the RNA-seq analysis (Methods, Additional file 1: Supplemental Table S10). If depletion of a vlincRNA has an effect on the genes it regulates, then the RNA levels of the negatively correlated genes should increase while those for the positively correlated ones, decrease (Fig. 6a). Thus, the fold changes of the former in response to vlincRNA knockdown would be higher than that of the latter. Conversely, if a vlincRNA has no effect on the genes it regulates, there should be no difference in the relative expression changes between genes negatively and positively correlating with it. To determine whether the difference exists, fold change of each gene was calculated for the 3- and 6-day time point relative to (1) the corresponding mismatch control and (2) the un-induced samples (the day 0 controls) based on RNA-seq analysis (Methods).

We then estimated differences in the relative fold changes between 4 groups of genes for every vlincRNA. The first 3 groups were based on the co-expressed genes: (1) all negatively vs all positively co-expressed genes, (2) 100 most negatively vs 100 most positively co-expressed genes, and (3) 50 most negatively vs 50 most positively co-expressed genes. The final background control group consisted of all remaining genes, many of which also exhibited weak correlation (either positive or negative) with vlincRNA expression, which however did not pass the significance thresholds described above for these genes to be considered co-expressed with vlincRNAs (Fig. 6a). In theory, the effect of vlincRNA depletion on these background genes should be less than on the co-expressed genes. Thus, the relative fold change difference in the background genes negatively and positively correlating with vlincRNA expression would serve as a control for the differences observed between the negatively and positively co-expressed genes (Fig. 6a). Therefore, the background group was split into two subsets based on negative or positive correlation with a vlincRNA and the differences in the relative fold changes between the two groups were calculated. For each comparison, we calculated 3 metrics: (1) differences between median relative fold changes, (2) Cohens *d* effects of differences between the average relative fold changes, and (3) statistical significance of the difference using the Wilcoxon rank sum test (Fig. 6be, Additional file 1: Supplemental Table S10). The comparisons were done by treating the 3- and 6-day time points separately and by combining the two time points.

Strikingly, the relative fold changes of the negatively co-expressed genes were almost always higher than those of the positively co-expressed ones as signified by the differences of the medians and positive Cohens *d* scores (Fig. 6be, Additional File 1: Supplemental Table S10). However, the differences of the medians and the Cohens *d* values were much higher for the co-expressed genes compared to the background correlated genes (Fig. 6be, Additional file 1: Supplemental Table S10). This difference was particularly pronounced when the top 50 or 100 negatively co-expressed genes were compared with the top 50 or 100 positively co-expressed ones (Fig. 6be, Additional file 1: Supplemental Table S10). Overall, the magnitudes of the Cohens *d* effects were quite small, mostly <0.1 for the control background genes (Fig. 6b, d, Additional file 1: Supplemental Table S10). The differences of the medians and Cohens *d* values were higher on the day 3 compared to day 6 (Fig. 6d, e, Additional file 1: Supplemental Table S10), possibly due to accumulation of indirect effects affecting expression of the target genes. Furthermore, using the Wilcoxon rank sum test, the median relative fold changes of the negatively co-expressed genes were significantly higher (*p* value <0.05) than those of the positively co-expressed ones for 2 out of 3 gRNAs (Additional file 1: Supplemental Table S10). However, several comparisons for the remaining gRNA were reaching the threshold of significance with the *p* values in the 0.050.09 range (Additional file 1: Supplemental Table S10). Based on these results, we reached the following conclusions. First, vlincRNAs appear to directly regulate multiple other genes, both positively and negatively, and these regulatory interactions could be predicted based on expression correlation in our co-expression assay. Second, genes with stronger co-expression with vlincRNAs do exhibit stronger regulation by the vlincRNAs. Third, even relatively modest levels of depletion of these transcripts can have measurable molecular phenotypes.

### Functional properties of vlincRNA regulatory networks

**Fig. 7:**
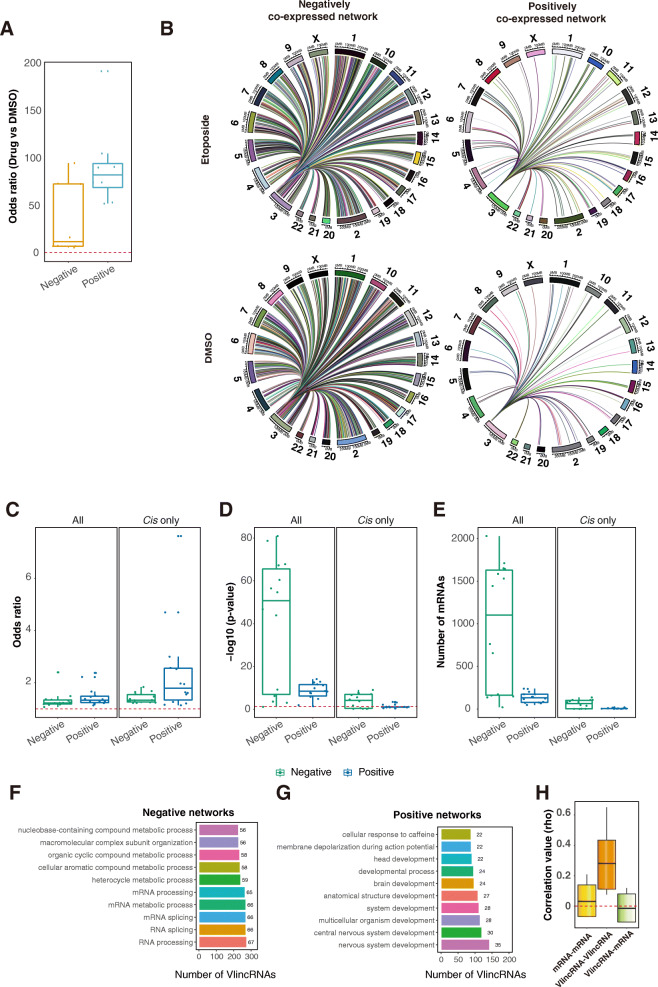
Properties of vlincRNA regulatory networks. **a** Stability of the regulatory networks in different treatmentsbox plots of the odds ratios of the overlap between networks in the DMSO- and drug-treated samples for the 6 vlincRNAs. **be** Regulation of multiple genes in *trans* and *cis*. Most of the genes in the positively and negatively correlating networks are found on different chromosomes as illustrated for the co-expression networks of the vlincRNA ID-1202 in either etoposide or DMSO treatments. Connections between the vlincRNA located on the chromosome 3 and each gene co-expressed (either positively or negatively) with it and containing site of vlincRNA-chromatin interactions are shown by the thin lines. Box plots of the odds ratios (**c**), *p* values (**d**), and the total number of genes in common (**e**) based on the comparisons of the co-expression networks and chromatin interaction datasets for either all genes (left plots) or genes found on the same chromosome (right plots) for the 14 vlincRNA-drug combinations. **f**, **g** Top ten GO terms enriched in genes found in either negative (**f**) or positive (**g**) co-expression networks for all 407 vlincRNAs. The GO terms were ranked based on the number of vlincRNAs (*X*-axes) whose networks were enriched in these terms. The numbers next to each term represent % of vlincRNAs containing the term out of the total 407 vlincRNAs. **h** Boxplots of the Spearman correlation values of all possible pairwise combinations of mRNA-mRNA, vlincRNA-vlincRNA, and mRNA-vlincRNA

The strong statistical overlap between the SMS co-expression and the chromatin interaction datasets combined with the CRISPR/Cas13 validation indicated we identified true vlincRNA regulatory networks. As described above, RAT signal for the genes in vlincRNA networks was significantly higher than in the background genes in most treatments. Therefore, it appears that different treatments did not significantly alter vlincRNA regulatory networks. To further quantify this observation, we identified lists of genes shared by the co-expression and chromatin interaction datasets in each treatment (DMSO or drugs) for each vlincRNA. Then, we estimated the fraction of overlap among these lists for each vlincRNA. Overall, 83.7100% (median of 92.9%) and 48.683.8% (median of 63.7%) of respectively negatively and positively correlated co-expressed genes were shared by the DMSO-treated controls and the drug treatments. The respective odds ratios of positive and negative co-expressions were 52.8192.7 (median 83) and 6.395.6 (median 12.6), indicating that the overlaps between the drug treatments and DMSO treatments were statistically significant (Fig. 7a, Additional file 1: Supplemental Table S11). Also, the networks did not change significantly in response to treatments with different drugs. For the two vlincRNAs profiled in both the etoposide and SN-38 treated cells, 87.299% (median of 93.1%) and 80.493.7% (median of 87.1%) of respectively negatively and positively correlated co-expressed genes were shared by the two drugs.

Second, networks consisted primarily of genes located on chromosomes different from those where the vlincRNAs were found, as exemplified in the Fig. 7b. However, consistent with the results above, the odds ratios of the overlap between the co-expression and chromatin interaction datasets were higher for the genes located on the same chromosomes (*cis*) as the vlincRNAs than those on the other chromosomes (*trans)* (Fig. 7c), but only 12/28 of these overlaps were statistically significant (Fig. 7d, Additional file 1: Supplemental Table S12). The likely reason for it is that the number of genes on the same chromosomes was not as high as genome-wide (Fig. 7e, Additional file 1: Supplemental Table S12). Therefore, we combined all samples to increase the statistical power and could indeed show that the odds ratios of overlap between the co-expression and chromatin interaction datasets were higher in *cis* than in *trans* (*p* value 6.1E3, Wilcoxon rank sum test). Therefore, these results suggest that vlincRNAs participate in both *cis* and *trans* interactions; however, while the latter are much more numerous, the RNA-chromatin interactions with the genes on the same chromosomes tend to be stronger (see the Discussion section).

To further understand the properties of the vlincRNA regulatory networks, we performed Gene Ontology (GO) analysis to annotate all 407 vlincRNAs based on the functions of genes in the networks. Strikingly, the networks for different vlincRNAs exhibited enrichment of similar functions (Fig. 7f, g, Additional file 1: Supplemental Table S13). Most of the negatively correlated networks were significantly enriched in functions related to RNA (Fig. 7f), while the positive networks were significantly associated with various development GO terms (Fig. 7g). For example, the top 5 enriched GO terms among the negatively correlated networks and shared by 65% of the vlincRNAs were RNA processing, RNA splicing, mRNA splicing, mRNA metabolic process, and mRNA splicing (Fig. 7f, Additional file 1: Supplemental Table S13). DNA-templated transcription was within top 20 such GO terms and shared by 50% of all vlincRNAs (Additional file 1: Supplemental Table S13). On the other hand, the top 5 GO terms enriched in the positively correlated networks were nervous system development, central nervous system development, multicellular organism development, system development, and anatomical structure development shared by 2735% of the vlincRNAs (Fig. 7g, Additional file 1: Supplemental Table S13). Extending to the top 50 GO terms revealed additional enrichment of the negatively co-expressed networks in functions associated with cell-cycle, such as cell cycle phase transition, cell cycle, mitotic cell cycle process, mitotic sister chromatid segregation, and negative regulation of cell cycle process shared by 4346% of vlincRNAs (Additional file 1: Supplemental Table S13). The same step revealed the enrichment in functions associated with cellular adhesion among the genes found in the positively correlated networks and shared by 1321% of vlincRNAs (Additional file 1: Supplemental Table S13).

All in all, the enrichment of similar functions among the co-expressed genes suggested that vlincRNAs have somewhat similar patterns of expression. To address this, we calculated median Spearman correlation among vlincRNAs or mRNAs only and between pairs of vlincRNAs and mRNAs. The median vlincRNA-vlincRNA, vlincRNA-mRNA, and mRNA-mRNA correlations were respectively 0.28, 0.02, and 0.03 (Fig. 7h). Thus, indeed vlincRNAs tend to be coordinately regulated and participate in control of genes with similar functions.

### VlincRNAs are required for cellular survival under stress conditions

To directly test whether vlincRNAs and their regulatory networks could have biological significance, we tested importance of the 2 vlincRNAs used for the CRISPR/Cas13 experiments for the cells ability to survive genotoxic stress. Cells from the 8 individual CRISPR/Cas13 cell lines described above were mixed in equal proportions, grown for 3days in presence or absence of Dox and then treated with etoposide (also with or without Dox). As shown in our previous study, etoposide had a strong and long-lasting toxic effect on K562 cells, leading to a continuous cell death even after the removal of the drug, and a slow recovery [22]. Here, for every treatment, after removing etoposide, the cells were allowed to regrow for ~10days until they resumed normal growth and appearance, and then we estimated survival of the cells harboring each gRNA by calculating the normalized abundance of that gRNA in the genomic DNA from the pooled cells using NGS. For every treatment and every gRNA pair, we calculated the ratio of targeting/non-targeting gRNA abundances to estimate relative survival of cell harboring gRNAs targeting vlincRNAs relative to cells harboring their cognate non-targeting controls.

Interestingly, the average/median of the ratios of the targeting gRNAs relative to their cognate controls either immediately after pooling or growth for 3days before etoposide addition were 0.9/0.91 even though all cell lines were mixed in equal proportions (Additional file 1: Supplemental Table S14, Methods). This suggests that even during growth and expansion steps leading from the lentiviral transfection to establishment of the individual cell lines, preferential loss of cells expressing targeting gRNAs occurred presumably due to their toxicity combined with the leaky expression of Cas13 in the absence of Dox. The subsequent treatments with etoposide resulted in further drop in this ratio, especially when combined with the induction of Cas13 by Dox (Additional file 1: Supplemental Table S14). The average/median ratios of targeting vs non-targeting gRNAs were 0.91/0.87 for the etoposide/Dox and 0.78/0.76 for the etoposide/+Dox treatment (Additional file 1: Supplemental Table S14). Overall, across all 4 gRNAs pairs, the drop in the ratio (indicating more dead cells) in the etoposide/+Dox samples was statistically significant with *p* values of 0.01 and 0.04 (one-sided Students *t* test) compared to the cells not treated with etoposide or those treated with etoposide/Dox. Interestingly, cells expressing the gRNA D33_v2_6 that did not show significant vlincRNA depletion in the transcriptome analysis were most depleted even without the drug treatment compared to their non-targeting control in the cell survival analysis with the corresponding average ratios of 0.84, 0.76 and 0.66 (Additional file 1: Supplemental Table S14). These results suggest that our inability to detect consistent changes in the level of the target transcript could be caused by the death of cells where this vlincRNA is depleted in a mixed population of cells. Altogether, these results demonstrate that these vlincRNAs are required for cell survival during normal growth conditions and especially so under a genotoxic stress.

### Effect of an RNA measurement platform on authenticity of co-expression derived networks

To test whether the significant overlap between the co-expression derived networks and chromatin interaction datasets would be a general feature for any expression dataset, we regenerated a fraction of the dataset used for the co-expression analysis using the 2nd generation Illumina platform also using rRNA-depleted total RNA. We generated the co-expression networks using the same criteria as above. Importantly, application of the same *p* value threshold to estimate the reliability of the correlation estimates should in theory account for the different numbers of samples used to calculate the co-expression correlations (64 for SMS vs 32 for Illumina). Furthermore, the Illumina dataset had a much larger (on average ~10 fold) number of reads generated per sample and significantly longer reads: paired-end 150 base reads vs single read of on average ~35 bases for SMS. For each vlincRNA, we found a higher number of co-expressed transcripts using the same thresholds in the Illumina RNA-seq dataset than in the SMS one with the corresponding median numbers of 2,073 and 1,615. As in the SMS-based co-expression analysis, we observed a statistically significant trend towards the negative correlation between vlincRNAs and mRNAs with the corresponding median numbers of negatively and positively co-expressed mRNAs of 1,119 and 943 per vlincRNAs (*p* value <2.2E16, Wilcoxon signed rank test).

However, the overlap with the RAT dataset was poor based on comparisons with individual vlincRNA-treatment combinations or on merged dataset made by combining all treatments for all vlincRNAs (Fig. 5e, f, Additional file 1: Supplementary Tables 15 & 16). While the odds ratios of the overlaps with the negatively co-expressed genes indicated enrichment and ranged from 1.03 to 1.74 (median 1.19) (Fig. 5e, Additional file 1: Supplemental Table S15), the *p* values were much less significant compared with those from SMS RNA-seq mentioned above, with the median *p* value of 3.7E2 (ranging from 2.22E47 to 0.35) (Fig. 5f, Additional file 1: Supplemental Table S15). Furthermore, the odds ratios for the positively co-expressed genes were much lower than those for the SMS RNA-seq dataset ranging from 0.8 to 1.06 with the median of 1.0 and the median *p* value being 0.53 (ranging from 3.09E2 to 0.93) (Fig. 3a, b, Additional file 1: Supplemental Table S15). Similar results were obtained using the merged data: while the overlap was significant for the negatively co-expressed vlincRNAs for both platforms albeit with the higher significance in the case of SMS, it was only significant for the positively co-expressed vlincRNAs detected by the SMS platform (Additional file 1: Supplemental Table S16). When both positively and negatively co-expressed vlincRNAs were combined, the overlap was only significant for the SMS platform (Additional file 1: Supplemental Table S16).

We also compared the effect of vlincRNA knockdown on the co-expression networks generated by both platforms. Overall, the Illumina-generated networks had similar profiles as the SMS ones (Additional file 1: Supplemental Table S17). However, the differences between the co-expressed genes in the networks and the background genes were mush less significant (Additional file 1: Supplemental Table S17). For example, the Cohens *d* effects of the combined day 3 and 6 data for the gRNAs gRNA D30_v6_6 and D33_v2_10 were 0.148 and 0.273 for the network genes and correspondingly 0.062 and 0.113 for the background genes for the SMS-generated networks (Fig. 6b, Additional file 1: Supplemental Table S10)on average 2.4-fold higher for the network genes. The corresponding values for the Illumina networks were 0.086 and 0.295 compared to 0.080 and 0.218 (Additional file 1: Supplemental Table S17)on average 1.2-fold higher. Therefore, networks generated using different expression platforms even on the same sample type would likely differ significantly in terms of their authenticity.

## Discussion

Here, we present a co-expression approach to functionally annotate lncRNAs belonging to the class of vlincRNAs based on a 3rd generation SMS. We further authenticate the vlincRNA regulatory networks derived using this approach with genome-wide maps of chromatin interactions and targeted knockdown of selected vlincRNAs. We provide several lines of evidence showing that the overlap between the co-expression networks and chromatin interaction dataset is not random and likely represents biologically meaningful interactions. First, the chromatin interaction profiles are reproducible in different biological replicas. Second, the signal derived from the chromatin interaction profiles is much stronger for the co-expressed genes compared to the background genes. Third, the overlap between genes showing evidence of proximity to lncRNAs and the co-expression dataset is statistically significant. Fourth, the strength of the overlap is proportional to the stringency of the cutoff used in the RAT assay. Finally, we provide direct proof that vlincRNAs regulate the target genes identified using the co-expression analysis via knockdown of specific vlincRNAs with the CRISPR/Cas13 system.

Furthermore, we show that the transcriptome analysis platform is crucial for obtaining authentic co-expression dataset. Usage of a platform requiring PCR amplification during library preparation step significantly reduced or totally abolished the concordance between the co-expression and the chromatin interaction datasets. This was especially apparent in correlation between vlincRNAsa relatively low abundant class of transcriptsand low-abundant mRNAs and was in agreement with the previous reports showing that SMS is more accurate in detection and quantitation of low-abundant transcripts compared to the platforms utilizing PCR amplification during library preparation such as Illumina and others [25]. While SMS has been used previously to generate a broad expression dataset across multiple cell type based on CAGE (cap analysis gene expression) technology by the FANTOM5 consortium [38], to our knowledge, no extensive SMS dataset based on RNA-seq and generated on a single cell type exists. Most lncRNA networks generated so far are based on co-expression analyses where expression measurements include PCR amplification steps during the library preparations. Furthermore, none of these networks have been independently validated. Therefore, the results presented here represent the first effort to generate lncRNA networks using cross-validation by independent molecular biological and reverse genetics approaches.

Altogether, in this work, we provide a guideline for a co-expression annotation strategy that could be broadly applied to annotating transcripts of unknown function, both coding and non-coding. Based on our results, such strategy would include the following features: (1) it should be based on a highly accurate whole-genome expression analysis that does not involve PCR amplification steps, (2) include short time of transcriptome perturbations to capture direct interactions, and (3) limited to a single cell type. However, it is also quite conceivable that strategies that can at least partially overcome PCR-induced artifacts such as unique molecular identifiers (UMI) might also work well in annotation strategies based on co-expression analysis.

Using this general strategy, we reach the following conclusions about functionality of the vlincRNA class of transcripts. First, vlincRNAs appear to regulate multiple genes both in *trans* and *cis*. In this respect, they resemble transcription factors, yet their mode of function is likely to be quite different. Second, even though the majority of genes regulated by vlincRNAs lie on different chromosomes, genes located in *cis* are more likely to be regulated by vlincRNAs. Third, vlincRNAs appear to mostly negatively regulate other genes with the exception of those in their immediate vicinity. This property would be similar to other lncRNAs reported to function by targeting repressive chromatin modifying components to promoters of their target genes [4, 5].

Fourth, the RNA-chromatin interaction assay used in this study does not require direct interaction between lncRNAs and the target DNA, but rather relies on their relative proximity in the nucleus. Recently, a number of experimental approaches assaying either direct interactions or immediate proximity between DNA and RNA molecules genome-wide have been reported [35,37]. However, our results suggest that lncRNAs also engage in functionally important *trans* interactions that are more distal to their target genes yet still having impact on regulation of expression of these genes. In this respect, it is noteworthy that vlincRNAs were previously reported to localize to discrete subnuclear structures [19, 29]. Together with these localization properties, the results presented here suggest that vlincRNAs might function by participating in formation of subnuclear domains and thus control expression and/or processing of multiple genes within these domains. Consistent with these results, lncRNAs were previously hypothesized to function as intelligent scaffolds that fulfill the role of organizers of micro-domains within nucleus that facilitate information exchange and computation necessary to deal with staggering complexity of molecular decisions taking place within the nucleus of a human cell [39]. Indeed, a precedent for this exists, as exemplified the *Neat1* lncRNA shown to represent a critical component of the subnuclear structures paraspeckles [40, 41]; however, our results suggest this functionality of lncRNAs might be much more common.

Interestingly, we found that any given vlincRNA can apparently regulate multiple (from hundreds to thousands) genes. This raises a question of how a lncRNAeven a very long onecould be in proximity of and affect so many genes. One possible explanation is that genes are enriched near vlincRNAs in the 3D organization of the nucleus, and this enrichment is captured by the RAT assay. In fact, *NEAT1* and *MALAT1* lncRNAs have been shown to not only form subnuclear domains but also interact with hundreds of genes [42]. Similarly, vlincRNAs could also provide connections between the multiple genes they interact with and the nuclear domains they help to form. Another possibility is that not all of these interactions take place in the same cell and the batch-based assays used in this study capture the full complexity of interactions happening in multiple cells. However, additional studies, likely using single-cell approach are needed to address this question.

Interestingly, all 407 vlincRNAs representing ~2% of the genomic space (similar to what is represented by exons of mRNAs) have similar patterns of expression and appear to regulate genes involved in relatively few types of functions. The latter include those related to various aspects of RNA metabolism, nervous system development, and development in general and, to a lesser extent, cell-cycle and cellular adhesion. Previously, the functions related to early development and early brain development were also found among positively correlating functions for vlincRNAs regulated by pluripotency-associated transcription factors Sox2, Nanog, or Oct4 using co-expression across multiple cell types [29]. Our data suggest that this association may be a more general feature of vlincRNAs and not necessarily limited to the ones regulated by the pluripotency-associated transcription factors.

The enrichment of cell-cycle related functions was also found previously among the genes negatively correlated with vlincRNAs [29]. Furthermore, the two characterized vlincRNAs*VAD* and *ASAR6-141*have been implicated in respectively control of cell cycle [19] and chromosomal replication timing [20]. It thus appears that regulation of cell cycle related functions might indeed be a common feature of vlincRNAs. Overall, networks consisting of genes encoding RNA-related functions and those involved in the cell-cycle fit quite well the potential involvement of vlincRNAs in DNA damage response found in this work. The cell cycle control is a major and well-understood component of this process [43].

However, perhaps the most striking and novel outcome of this study is the association of the majority of vlincRNAs with regulating genes encoding functions involved in RNA processing and metabolism. The complexity of transcriptome in a human cell is staggering with almost every gene possessing alternative isoforms due to initiation, termination and splicing with many such isoforms still undiscovered [44, 45]. Regulation of this complexity must itself be extremely complex and dynamic to respond to the various environmental and developmental cues and challenges faced by a cell. Furthermore, DNA damage response is known to involve multiple genes whose products function in RNA splicing and RNA metabolism [46]. The results presented here argue that vlincRNAs could provide novel regulatory hubs responsible for coordination of response of cells to DNA damage predominantly via regulating genes encoding RNA-related functions. In general, the vlincRNAs appear to serve as regulators of the regulators of the transcriptome and thus potentially represent an important component of the hidden layer of RNA-based control postulated to occur in cells of complex organisms [10, 11].

## Conclusions

This work represents the first comprehensive study of regulatory networks uncovered for a novel class of lncRNAsvlincRNAs. The networks were initially obtained using the co-expression analysis based on SMS transcriptome profiling and then validated using two different approaches. This cross-validation approach confirmed the authenticity of these lncRNA regulatory networks and ensured validity of the following major conclusions derived from the analysis of these networks. First, a typical vlincRNA appears to function by regulating expression of multiple genes in *cis* and *trans*. Second, a vlincRNA can have both positive and negative effects on expression of different target genes. Third, vlincRNAs tend to regulate genes encoding certain functions related most notably to RNA processing, cell cycle, development, and adhesion. Fourth, the regulation depends on a mechanism based on co-localization, yet not necessarily direct interaction, of the vlincRNAs and their target genes in nucleus. In this regard, two other well-characterized lncRNAs *NEAT1* and *MALAT1* show similar mode of regulation. It is tempting to speculate therefore that regulation of multiple genes and doing so via proximity in the nucleus might be a common mode of lncRNA functionality. Furthermore, targeted knockdown of 2 vlincRNAs in stable cell lines has revealed biological importance of these transcripts and their regulatory networks in survival in response to genotoxic stress at least at the level of cultured cells similar to the results obtained previously by our group in a high-throughput screen [22]. However, additional studies are required to address of functionality of vlincRNAs, and lncRNAs in general, at the organismal level [12].

## Methods

### Biological material and reagents

Human CML leukemia cell line K562 was obtained from Cell Bank of Chinese Academy of Sciences. Cells were cultured in RPMI 1640 (Thermo Fisher Scientific) supplemented with 10% (v/v) heat-inactivated fetal bovine serum (Thermo Fisher Scientific) and 1% (v/v) pen-strep (Thermo Fisher Scientific) at 37C and 5% CO_2_. For drug treatments, 1.5 million cells were seeded in 3ml of the medium without antibiotic per well in 6-well plates. After 16h, various drugs or DMSO/water controls were added at the concentrations indicated in Additional file 1: Supplemental Table S1 and incubated for 3 or 6hr. All drugs were obtained from Abmole Bioscience Inc. and dissolved in DMSO (with the exception of YM-155 and cytarabine that were dissolved in water) (Additional file 1: Supplemental Table S1). The concentration of DMSO was kept at 0.1% in all treatments. Total RNA was extracted using TRNzol Universal (Tiangen, DP424) and Total RNA kit I (Omega, R6834-02) according to the manufacturers protocols and quantified using Qubit 3.0 Fluorometer (Life technologies). Total RNA (5g) was treated with 5U of TURBO DNase (Thermo Fisher Scientific, AM2238) in 1 TURBO DNase buffer (Thermo Fisher Scientific, AM2238) at 37C for 30min to remove genomic DNA, and then purified with 2 volumes of VAHTS RNA Clean Beads (Vazyme, N412). The DNase-treated material was subjected to either RNA-seq or real-time qPCR analysis.

### RNA-seq

#### Helicos/SeqLL SMS

cDNA synthesis was performed with a modified version of the NSR hexamer method to avoid synthesis of rRNA. DNase-treated RNA (1g) was combined with 80ng of a mixture of 749 NSR hexamers (described by [28] and synthesized by Shanghai TranSheep Bio Co. Ltd), 4l of 10mM dNTP and water to a total volume of 13l. The mix was heated at 65C for 5min and kept on ice before adding 4l of 5 SuperScript III reaction buffer and 1l of 0.1M DTT followed by incubation at 25C for 20min. The sample were moved on ice and 1l of SuperScript III enzyme (Invitrogen) was added followed by incubation at 25C for 10min, 40C for 40min, 55C for 50min, and 75C for 5min. The RNA template was removed by adding 1l of RNase H (New England Biolabs Inc.) and 1l of RNase If (New England Biolabs Inc.) and incubating at 37C for 30min. cDNA was purified using Performa DTR Gel Filtration Cartridges (EdgeBio) and polyAtailed with TdT essentially as previously described [2]. Sequencing was outsourced to the SeqLL, LLC facility (Woburn, MA, USA).

#### Illumina

After DNase-treatment, the total RNA samples were used for RNA-seq library construction using rRNA-depletion strategy. The library construction and the Illumina sequencing using paired end 150bp strategy on 10-gigabase (GB) scale was outsourced to Novogene Corporation (Beijing).

### Real-time qPCR analysis

The DNase-treated RNA (1g) was used for first-strand cDNA synthesis using SuperScript III kit (Invitrogen, 18080093) and 80ng random hexamers according to the manufacturers protocols. The cDNA was purified with 2 volumes of VAHTS DNA Clean Beads (Vazyme, N411). The concentrations of the cDNA samples were always measured on Qubit 3.0 fluorometer (Life Technologies, MAN0010876) with Qubit ssDNA Assay kit (Invitrogen, Q10212). Then, either 10ng or 60ng cDNA was combined with 5l PowerUp SYBR Green Master Mix (Thermo Fisher Scientific) and 0.5l of each primer (10M) in total volume of 10l. Each primer pair-sample combination was represented by 3 independent technical qPCR replicas. The Ct values were obtained using MxPro software (Agilent Technologies, Inc) with Comparative Quantitation (Calibrator) settings.

### RAT assay

#### Crosslinking and cell lysis

For 4 vlincRNAs (ID-1132, ID-1205, ID1299 and ID1274) (Additional file 1: Supplemental Table S18) found to be induced only by etoposide, the RAT procedure was performed on nuclei isolated from cells treated with either etoposide or DMSO. For 2 vlincRNAs (ID-1202 and ID1107) (Additional file 1: Supplemental Table S18) induced by either etoposide or SN-38, the RAT procedure was performed on nuclei isolated from cells treated with either etoposide, SN-38 or DMSO.

K562 cells (510^5^ cells/ml) were incubated in RPMI 1640 (ThermoFisher Scientific) supplemented with 10% (v/v) fetal bovine serum (ThermoFisher Scientific) at 37C and 5% CO_2_ for 16h and treated with 1M SN-38 (Abmole Bioscience Inc, M3016) or 100M etoposide (Abmole Bioscience Inc, M2326) or the same volume of DMSO as control for 6h. The cells (3 10^6^) were crosslinked in 1ml of growth medium supplemented with 1% formaldehyde for 10min at room temperature and immediately collected by centrifugation at 1000rpm for 10min at 4C. Cells were washed twice with 5ml cold 1 PBS followed by treatment with 100l of 1.375M glycine to neutralize crosslinking at room temperature for 2min and centrifugation at 3500rpm for 15min at 4C. To prepare the nuclei, the crosslinked cells were lysed with 5ml cell lysis buffer (1mM Tris [pH8.0], 10mM NaCl, 0.2% NP-40, 1 protease inhibitors (Abmole Bioscience Inc, M5293)) for 1h at 4C with mixing and centrifuged at 2500rpm for 15min at 4C followed by lysis for additional 15min. The nuclei were washed twice with 500l 1 first strand reverse transcription buffer (FS buffer: 50mM Tris-HCl (pH8.0), 75mM KCl, 3mM MgCl_2_) and suspended in 100l 1 FS buffer (Invitrogen) in the presence of 0.3% sodium dodecyl sulfate (SDS) and incubated at 37C for 1h with gentle mixing. In order to sequester the SDS, triton X-100 was added to a final concentration of 1.8%. Nuclei were centrifuged at 2500rpm for 15min at room temperature and washed twice with 500l 1 FS buffer.

#### Strand-specific reverse transcription (SSRT)

The nuclei were resuspended in 80l SSRT solution (9l primer set containing 5M of each oligonucleotide, 3.75 l of 0.4mM biotin-14-dCTP (Invitrogen), 6 l of 10mM dNTP (Invitrogen), 800U Maxima Reverse Transcriptase (Thermo Fisher Scientific), 160U RNaseOUT (Invitrogen), 6 l of 0.1M DTT (Invitrogen) and 1 FS buffer (Invitrogen)) and incubated at 60C for 50min, followed by heating at 85C for 5min to inactivate the enzyme. For each vlincRNA, we designed 2 sets of specific non-overlapping oligonucleotides complementary to the RNA sequence of each of the six vlincRNAs mentioned in the text (Additional file 1: Supplemental Table S18). The oligonucleotides were separated by ~5kb and pooled together as primer set to a final concentration of 5M for each. Separate SSRT reactions performed in parallel with the two specific oligonucleotide sets and without the oligonucleotides as the control. All RAT experiments were done in two biological replicas.

#### Chromatin DNA fragmentation

After SSRT, samples were washed twice with 500 l 1 NEBuffer 3 (New England BioLabs). For chromatin fragmentation, samples were digested with 250l 1 NEBuffer 3 containing 250U DpnII (New England BioLabs), 250U AluI (New England BioLabs) and 250U MseI (New England BioLabs) for 6h at 37C.

#### Size detection of fragmented chromatin

Fragmented chromatin particles were collected and resuspended in the sorting buffer (2 saline-sodium citrate, 20% (vol/vol) formamide and 0.2mg/ml RNase-free BSA in nuclease-free water) and detected based on SSC (side scatter) and FSC (forward scatter) under 488nm excitation using the MoFlo Astrios EQ flow cell sorter (Beckman Coulter). Latex beads measuring 100, 200, and 300nm from the Photon Correlation Spectroscopy control mixed kit (Beckman Coulter, 6602336) were used as the standard size particles, and the sorting buffer was used as the background control. The data was collected and analyzed by the Summit 6.2 software (Beckman Coulter), and the gates were set based on the standard particle sizes. The measurements were done in 3 biological replicas with one shown in Fig. 3c. On average, ~250 and ~ 480 thousand particles were measured for the buffer-alone control and the fragmented chromatin samples respectively.

#### Enrichment and crosslink reversal

Pierce Streptavidin Magnetic Beads (ThermoFisher Scientific) were washed with binding/wash buffer (25mM Tris, 0.15M NaCl, pH7.2, 0.1% Tween-20) following the manufacturers instructions. Each sample was mixed with 20l pre-washed magnetic beads and incubated at room temperature for 2h with mixing to enrich the complexes containing vlincRNA/biotinylated-cDNA/chromatin DNA. The beads were collected by magnetic stand and washed six times with 300 l of the binding/wash buffer. The crosslink reversal was performed by incubation with 50 l binding/wash buffer and 50 l of 20mg/ml proteinase K (Invitrogen) overnight at 65C. After collecting the beads with a magnetic stand, the supernatant was saved and used for the subsequent steps.

#### DNA extraction

To remove RNA, 50U RNase If (New England BioLabs) was added into the supernatant and incubated at 37C for 1h. DNA was harvested in following steps. First, the sample was vigorously mixed with the same volume of phenol-chloroform-isoamyl alcohol (25:24:1, v/v) and centrifuged at the top speed for 5min at room temperature. Second, after removing the colorless upper aqueous phase into a new tube, the DNA was precipitated by adding 2.5 volume of absolute ethanol, 0.1 volume of 5M NH_4_Ac and 20g of glycogen (ThermoFisher Scientific) and incubating for 30min at 80C followed by centrifugation at the top speed for 10min at 4C. Third, the pellet was washed with 1ml ice-cold 70% ethanol and vacuum-dried, followed by resuspension in 22 l of water. Fourth, the concentration of the DNA was measured using Qubit 3.0 fluorometer and dsDNA HS Assay kit (Thermo Fisher Scientific).

#### NGS library preparation and sequencing

The libraries were constructed with the NEBNext Ultra DNA Library Prep Kit for Illumina (New England BioLabs) following the manufacturers recommendations and sequenced on the Illumina HiSeq X Ten platform using paired end 150 base strategy by the Novogene Corporation (Beijing) and 10-GB of raw data was collected for each sample.

### Construction of the vlincRNA inducible knockdown cell lines and their transcriptomic and phenotypic analyses

#### Construction

Two pairs of targeting gRNAs and corresponding mismatch controls with mutations in the bases 1214 of the 28-mer gRNAs (Additional file 1: Supplemental Table S18) [22] were selected for each of the two vlincRNAs ID-1162 and ID1107 corresponding to vlincRNAs ID-838 and ID274 used by Xu et al [22] and cloned into the gRNA vector pLentiguide by Golden Gate cloning. Lentivirus particles were produced by transfecting the 293FT packaging cell line with plasmid expressing each individual gRNA and used to transfect the TRE-LwCas13a-K562 cell line expressing Dox-inducible Cas13 [22]. Transfected cells stably expressing the gRNA sequences were selected by flow cytometry (BD CytoFLEX) using mCherry as the selection marker to generate vlincRNA inducible knockdown cell lines. These cell lines were generated by SyngenTech (Beijing, China).

#### Transcriptome analysis

For the expression analysis of the vlincRNA knockdown cell lines, cells (5 10^5^ cells/ml) were grown in 10ml culture medium supplemented with 1g/ml Dox for 0, 3 and 6days. RNA was isolated with E.Z.N.A. Total RNA Kit I (Omega) and RNA-seq was performed on the Illumina NovaSeq platform by the Novogene Corporation (Beijing, China) using rRNA-depletion protocol and paired end 150bp strategy on 10-GB scale.

#### Survival challenge experiment

For each vlincRNA ID-1162 and ID1107, 3.5 10^5^ cells from each of the 8 cell lines harboring individual targeting and corresponding mismatch control gRNAs (4 cell lines per vlincRNA) were pooled together and incubated in 10ml culture medium supplemented with 1g/ml Dox (or Dox control) in a T25 flask for 3days before seeding. Then, 7 10^5^ of mixed cells were seeded into a well of 6-well plates containing 2ml medium and treated with 25M etoposide (AbMole BioScience, USA) in the presence of Dox or water (Dox) control. After 24h, cells were collected, washed twice with 1ml RPMI 1640 medium to remove the drug and resuspended in 2ml fresh culture medium with Dox or without Dox for recovery. For each well, cells were passaged daily with the maximum density of 3.5 10^5^ cells/ml, until most cells recovered the normal shape and the doubling rate of the untreated cells. Genomic DNA was harvested from cells immediately after pooling, 3days after the Dox (or Dox control) treatment but before adding etoposide, and at the end of the survival challenge using TIANamp Genomic DNA Kit. The gRNAs were PCR amplified from the genomic DNA and profiled by NGS to estimate the abundance of each gRNA as described previously [22]. The gRNA profiles after survival challenges were analyzed to assess the effect of vlincRNA knockdown on the cell survival based on the abundance of cell harboring the targeting gRNAs compared to their cognate mismatch controls. Three independent biological repeats were performed for each drug treatment. In some treatments, an outlying replica was removed, resulting in a minimum of 2 biological repeats per treatment.

### Bioinformatics

The R environment was used for all bioinformatics analyses. Known genes were represented by the UCSC Genes database, specifically the table knownGene.txt downloaded from the GRCh37/hg19 assembly of the UCSC genome browser [47]. And for each gene, only the longest annotated transcript (based on the total length(s) of the exon(s)) was chosen for the subsequent analysis. The overlaps between genomic coordinates were calculated using the intersect function of the BEDTools suite (v2) [48]. The enrichment *p* values of overlaps were calculated using hypergeometric tests in the R environment. All Wilcoxon rank-sum tests mentioned in the text were two-sided, while Wilcoxon signed rank tests and hypergeometric tests were one-sided.

### RNA-seq data analysis of K562 cell line treated with anticancer drugs

#### Helicos/SeqLL SMS

Raw SMS reads were filtered for length ( 25 bases) and sequence quality using Helisphere package as previously described [2]. The remaining reads were then aligned to the GRCh37/hg19 assembly of the human genome using indexDPgenomic software [49]. RPKM values were calculated for each transcript annotated in UCSC Genes database based on reads mapping to exons of these transcripts and vlincRNAs as previously described [2].

#### Illumina

(1) Only paired end raw reads where each read was 30 bases after adaptor trimming and each base had the Phred quality score 20 were selected. (2) Such reads were then aligned to the GRCh38/hg38 assembly of the human genome by the Tophat software [50], and the uniquely mapping reads were used to calculate the FPKM for each transcript annotated in UCSC Genes database and vlincRNAs by the Cufflinks software with the default parameters [51]. Only the genes shared with the annotation used for the *Helicos/SeqLL SMS* analysis were kept for further analysis.

### Co-expression analysis

Spearman correlations were calculated between each vlincRNAs and a protein-coding mRNA using the stats.spearmanr function from the NumPy package in Python for the SMS and Illumina RNA-seq datasets separately. Only the pairs with the correlations >0.35 or < 0.35 and the corresponding *p* values <0.01 were used for the subsequent analyses.

### RAT data analysis

#### Generation of the average normalized RAT score

1. Only paired end raw reads where each read was 30 bases after adaptor trimming and each base had a Phred quality score 20 were selected. Such reads were then aligned to the GRCh37/hg19 assembly of the human genome using the BWA-MEM (Version 0.7.12) aligner with the default settings. Only the pairs of the reads where both paired reads uniquely mapped to the genome with the appropriate configuration and spacing were kept.
2. For each target oligonucleotide set and the no-oligo control, the alignments were converted into density format where each position of the genome was assigned score based on the total number of reads mapping there and normalized to the total number of reads in the sample.
3. For each target oligonucleotide set, the RAT signal from the step above was subtracted by the normalized signal of the corresponding control no-oligo treatment.
4. For each drug-vlincRNA combination, the score of RAT signal of each position in the genome was defined as the average of the control-subtracted normalized scores from the two RAT replicas.

#### Analysis based on ANARS (Fig. 4)

1. For each vlincRNA, all genes were divided into 3 groups: (i) positively co-expressed, (ii) negatively co-expressed, and (iii) the rest of genes was considered as background.
2. ANARS was derived as follows (Additional file 3: Supplemental Figure S2):
  - The length of each gene was normalized to 5kb resulting in the relative coordinates ranging from 1 to 5000 shown on Fig. 4a, d.
  - Finally, ANARS was calculated for each position (from 1 to 5000) of each group of genes defined in the step 1 by summing up the normalized average RAT score of such position and then dividing by the number of genes.
3. Furthermore, for each gene, the upstream and downstream 5kb regions were extracted and then corresponding ANARS values were calculated as in the step 2, but without the length normalization.
4. ANARS for each category of genes (co-expressed or background) in each sample was ranked and the top 30% were used for calculation of the significance of the difference between co-expressed genes and background control genes. Only non-zero ANARS values were used in the comparison between the *cis* co-expressed genes and all co-expressed genes. One-sided Wilcoxon rank sum test was used in this analysis.

#### Generation of RAT regions for each replica (Fig. 3d)

1. A threshold of >0 was applied to average normalized RAT score to generate regions where the signal of each base was higher in the specific oligonucleotide sample than in the no-oligo control.
2. The resulting regions were further filtered for precise overlap between the two oligonucleotide sets and only shared regions were extracted.
3. The resulting regions were further filtered based on the magnitude of the RAT signal after the subtraction of the no-oligo controls. The RAT signal for each base that survived filtration in the step 1 was ranked and thresholds based on the top 1%, 5%, 10%, 20%, and 30% of the signal were applied. Adjacent bases with signal above corresponding thresholds were merged as regions.
4. The genes containing within their boundaries such regions for each replica and each vlincRNA-treatment combination for each %-ile threshold were then selected and used in the analysis shown in Fig. 3d. For this analysis, the RAT regions within gene boundaries were defined either by a union (gene-level analysis) or by overlap of regions found in both replicas requiring precise matching of genomic coordinates (region-level analysis).

#### Analysis of overlap between genes containing RAT regions and co-expressed genes (Fig. 5)

1. Lists of genes based on the gene-level and region-level analyses were generated for each threshold and each vlincRNA-treatment combination.
2. The overlaps between co-expression networks and vlincRNAs-chromatin networks (Fig. 5ac) were calculated as follows:
  - For each mRNA, the maximum FPKM among the 64 SMS RNA-seq samples was calculated, and mRNAs with the maximum FPKM >0 were retained for the downstream analysis.
  - The mRNAs were ranked by the maximum FPKM values from largest to smallest, and the top and bottom 50% expressed genes were defined as either top half or bottom half mRNAs.
  - Based on the separation, the positively co-expressed genes were further divided into those derived from the top or bottom half of expression and the same was done for the negatively co-expressed genes resulting in 4 groups.
  - Each of the 4 groups of co-expressed genes for each vlincRNA was used to calculate the significance of overlap with the gene and region-level RAT signal for each treatment of the corresponding vlincRNA. The significance of the overlap was calculated separately for each RAT signal threshold with single-sided hypergeometric test (Fig. 5ac).
3. To build the final regulatory networks (Fig. 5d) for the 6 vlincRNAs using in the RAT analysis, RAT signal thresholds yielding lowest *p* values in the gene level analysis in the step 2d for the genes derived from the top half of expression for the negatively and bottom half for the positively co-expressed genes. The resulting networks were used in the analyses show in Fig. 7ae.
4. The same analytical steps were used to generate overlap between the RAT and co-expression results obtained using Illumina RNA-seq.

### GO analysis

The analysis was performed on positively and negatively co-expressed genes found using SMS RNA-seq for all 407 vlincRNAs as defined in the Co-expression analysis section. The gene set enrichment analysis was performed using the GOstats package (version 2.46) [52] in the R environment (package org.Hs.eg.db), and the *p* values adjusted according to the Benjamini and Hochberg approach were calculated using the package multtest with the parameter BH also in R. The GO terms with the adjusted *p* values <0.01 were further trimmed by the REVIGO software [53].

### RNA-seq analysis of the vlincRNA inducible CRISPR/Cas13 knockdown cell lines

The procedures of calculating FPKM for each transcript annotated in the UCSC Genes database and vlincRNA were identical to the Illumina RNA-seq data analysis described above. In order to test whether depletion of a vlincRNA has an effect on the genes it regulates, the following analytical steps were performed:

1. Genes with FPKM >1 in at least one sample were selected for downstream analysis.
2. For each gene or vlincRNA in each pair of cell lines expressing targeting or mis-match control gRNA, we calculated fold changes (FC) at days 3 or 6 of Dox treatment relative to the day 0 as follows: FC-T_3_ = T_3_/(T_3_ + T_0_), FC-T_6_ = T_6_/(T_6_+ T_0_), FC-NT_3_ = NT_3_/(NT_3_ + NT_0_), FC-NT_6_ = NT_6_/(NT_6_ + NT_6_), where T_0_, T_3_, T_6_, NT_0_, NT_3_ and NT_6_ are FPKM values for correspondingly the targeting (T) and mis-match (NT) gRNAs at days 0, 3, and 6.
3. We then calculated relative fold change (RFC) to estimate change relative to the control mis-match gRNA for each gene or vlincRNAs as follows: RFC-T_3_ = FC-T_3_/(FC-T_3_ + FC-NT_3_), RFC-T_6_ = FC-T_6_/(FC-T_6_ + FC-NT_6_), and the average RFC-T_3/6_ = (RFC-T_3_ + RFC-T_6_)/2.
4. For each gRNA, genes were separated into four groups based on the Spearman correlation (rho) of the co-expression with the targeted vlincRNA as follows: positively co-expressed genes (rho >0.35 and *p* value <0.01), negatively co-expressed genes (rho < 0.35 and *p* value <0.01), background negatively correlated control genes (rho <0.35 or *p* value >0.01), background positively correlated control genes (rho > 0.35 or *p* value >0.01). We then ranked the genes based on the Spearman correlation with the corresponding vlincRNA and extracted the top 50 and 100 significant positively and negatively correlated genes.
5. We then calculated the differences of medians and Cohens *d* effects between the corresponding groups of the negatively and positively correlated genes. We also used the Wilcoxon rank sum test to test whether the relative fold changes of genes negatively correlating with each target vlincRNA are larger than those positively correlating.

## Supplementary Information

**Additional file 1: Table S1.** Drugs used in SMS and Illumina RNAseq analyses. **Table S2.** DE mRNAs or vlincRNAs found in each drug treatment in the SMS RNAseq analysis. **Table S3.** Summary of the validation of the DE vlincRNAs by real-time PCR. **Table S4.** Numbers of mRNAs found to be co-expressed with each vlincRNA using either SMS or Illumina RNAseq. **Table S5.** Analysis of the concordancy of the two biological replicas (B1 and B2) of the RAT experiments. **Table S6.** Comparison of the aggregated RAT signal of different types of genes based on single-sided Wilcoxson Rank Sum Test. **Table S7.** The overlap between co-expression and the chromatin interaction datasets for top and botton half of expressed mRNAs. **Table S8.** Significance of the overlap between the co-expression and vlincRNA-chromatin interaction datasets with increasing RAT signal thresholds. **Table S9.** Overlap between the co-expression networks obtained after the separation based on the expression level and the chromatin interaction dataset for the SMS RNAseq platform. **Table S10.** Relative fold changes of the co-expressed transcripts in response to vlincRNA knockdown. **Table S11.** Genes in common between the co-expression and chromatin interaction datasets for each vlincRNA tend to be found consistently in drug and DMSO treatments. **Table S12.** The overlap between the co-expression and chromatin interaction datasets limited to the genes located on the same chromosomes based on top 50% and bottom 50% expressed mRNAs. **Table S13.** The top 100 GO terms enriched in the genes either negatively (Table S13.1) or positively (Table S13.2) co-expressed with the vlincRNAs. **Table S14.** Ratios of the normalized counts of each targeting gRNA vs the cognate non-targeting gRNA control in the mixed CRISPR/Cas13 cell lines subjected to various treatments. **Table S15.** Overlap between the co-expression networks and the chromatin interaction dataset for the Illumina RNAseq platform. **Table S16.** Overlap between the co-expression and the chromatin interaction datasets based on merged data for SMS and Illumina RNAseq platform. **Table S17.** The effect of vlincRNA knockdown on Illumina-based co-expression networks. **Table S18.** Coordinates of vlincRNAs and sequences of gRNAs used in the RAT and Cas13 knockdown cell lines.

**Additional file 2: Supplemental Figure S1.** UCSC Browser shot for the vlincRNA ID-1202 (Additional file 1: Supplemental Table S18) used in the RAT experiments. The vlincRNA (found on the minus strand) is antisense to RTP4 gene. K562 promoters are based on the Chromatin State Segmentation by HMM from ENCODE/Broad track. K562 nuclear polyA- RNAseq is based on the Long RNA-seq from ENCODE/Cold Spring Harbor Lab track. K562 nuclear polyA- CAGE is based on RNA Subcellular CAGE Localization from ENCODE/RIKEN track. The genomic strand is denoted as either (+) or (-). The gene RTP4 antisense to the vlincRNA is based on the UCSC Genes database. Zoom-in view of the boxed region in the panel (a) is shown in (b).

**Additional file 3: Supplemental Figure S2.** A flow chart diagram of calculating average normalized aggregated RAT score (ANARS) for gene boundaries and upstream or downstream 5kb flanking regions.

**Additional file 4: Supplemental Figure S3.** ECDF plots of ANARS in the boundaries and upstream or downstream 5kb flanking regions of positively and negatively co-expressed genes and background genes for each indicated vlincRNA-treatment combination.

**Additional file 5: Supplemental Figure S4.** Distribution of the statistical significance of enrichment of ANARS in the co-expressed vs the background genes (top) and *cis* only vs all co-expressed genes (bottom) for gene boundaries and upstream or downstream 5kb flanking regions among all indicated vlincRNA-treatment combinations. The *p* values were calculated based on the top 30% of the ranked ANARS values in each treatment as described in text and Methods.

**Additional file 6: Supplemental Figure S5.** Boxplots of non-zero ANARS in the cis and all co-expressed genes for gene boundaries and upstream or downstream 5kb flanking regions and for all indicated vlincRNA-treatment combinations.

## Data Availability

NGS data have been deposited to GEO with accession No: GSE173221 [54].
