# Supplementary figures and images for: Very long intergenic non-coding (vlinc) RNAs directly regulate multiple genes in cis and trans
Source: BMC Biol. 2021 May 20;19:108. doi: 10.1186/s12915-021-01044-x (PMC8139166; doi:10.1186/s12915-021-01044-x)

B

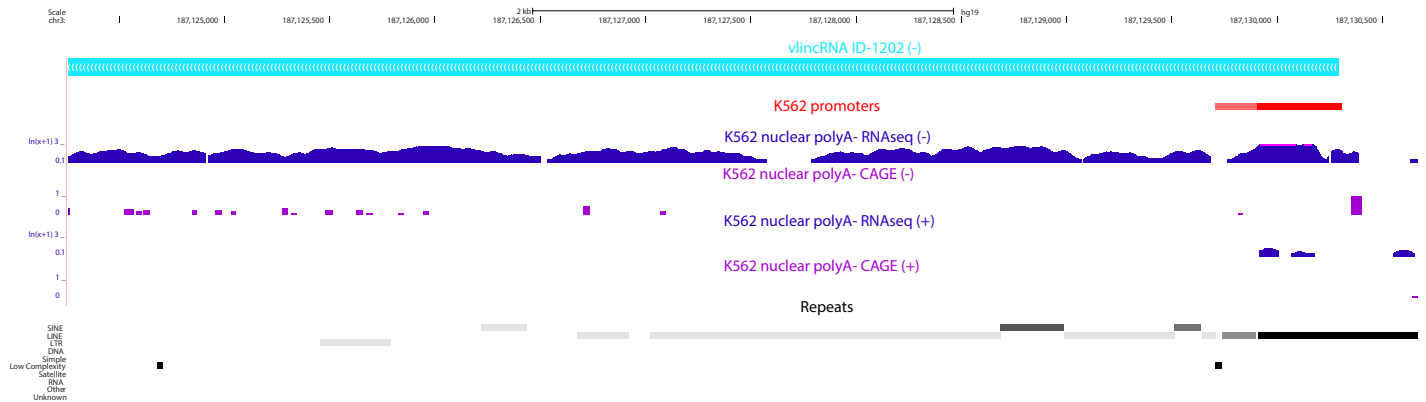

Supplement: Supplementary file 2 — Additional file 2: Supplemental Figure S1. UCSC Browser shot for the vlincRNA ID-1202 (Additional file 1: Supplemental Table S18) used in the RAT experiments. The vlincRNA (found on the minus strand) is antisense to RTP4 gene. K562 promoters are based on the Chromatin State Segmentation by HMM from ENCODE/Broad track. K562 nuclear polyA- RNAseq is based on the Long RNA-seq from ENCODE/Cold Spring Harbor Lab track. K562 nuclear polyA- CAGE is based on RNA Subcellular CAGE Localization from ENCODE/RIKEN track. The genomic strand is denoted as either (+) or (-). The gene RTP4 antisense to the vlincRNA is based on the UCSC Genes database. Zoom-in view of the boxed region in the panel (a) is shown in (b). [file 12915_2021_1044_MOESM2_ESM.pdf]

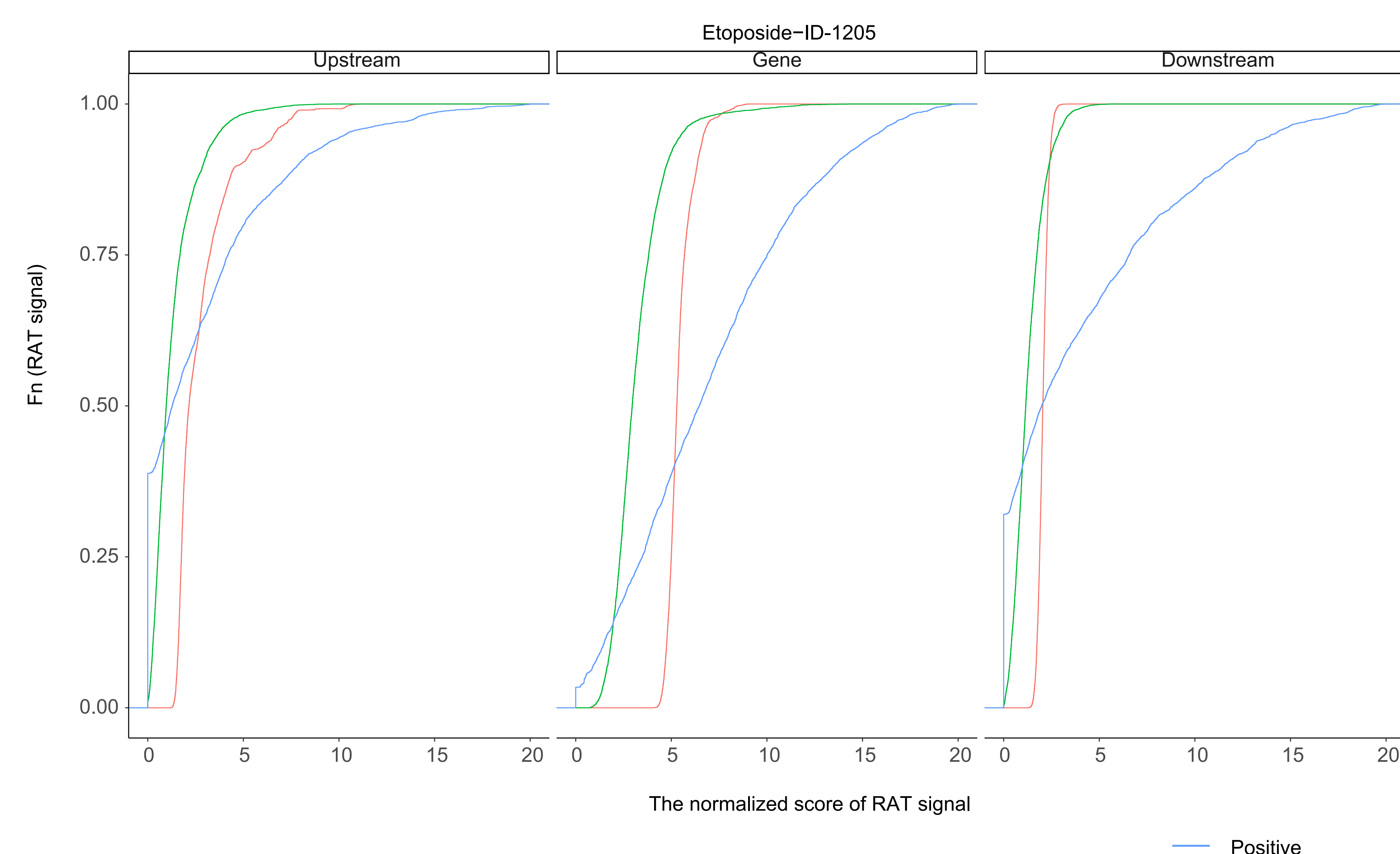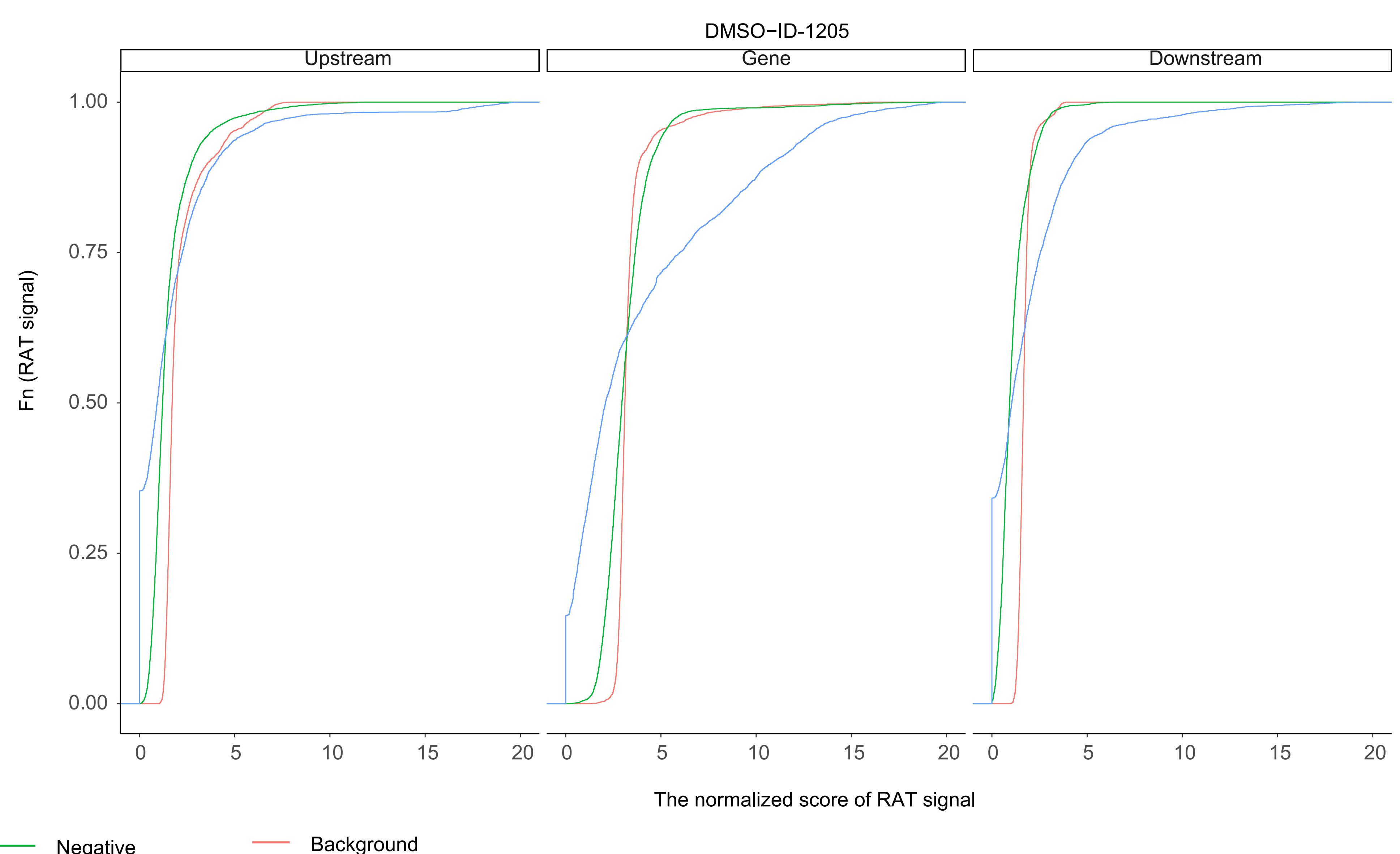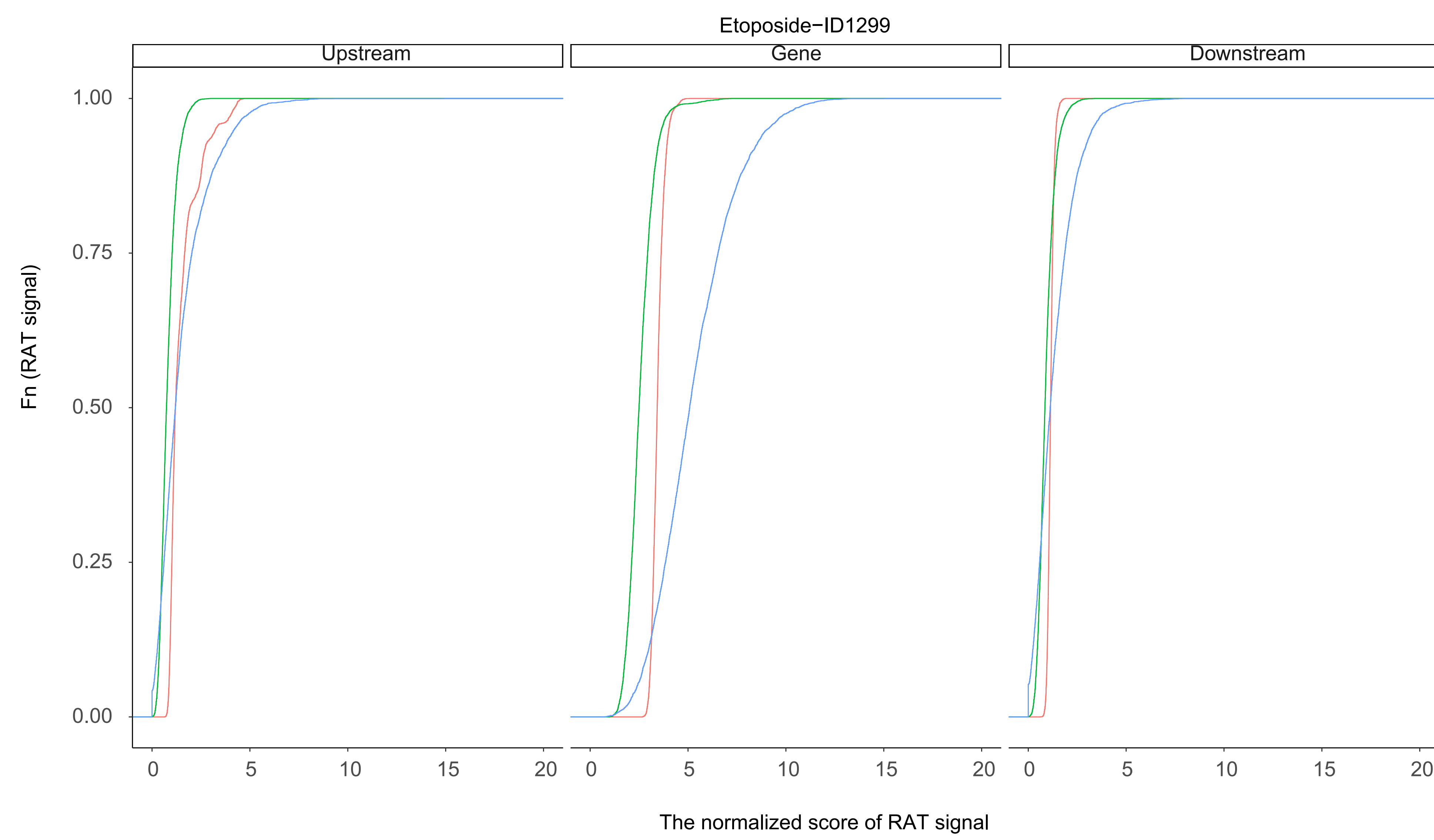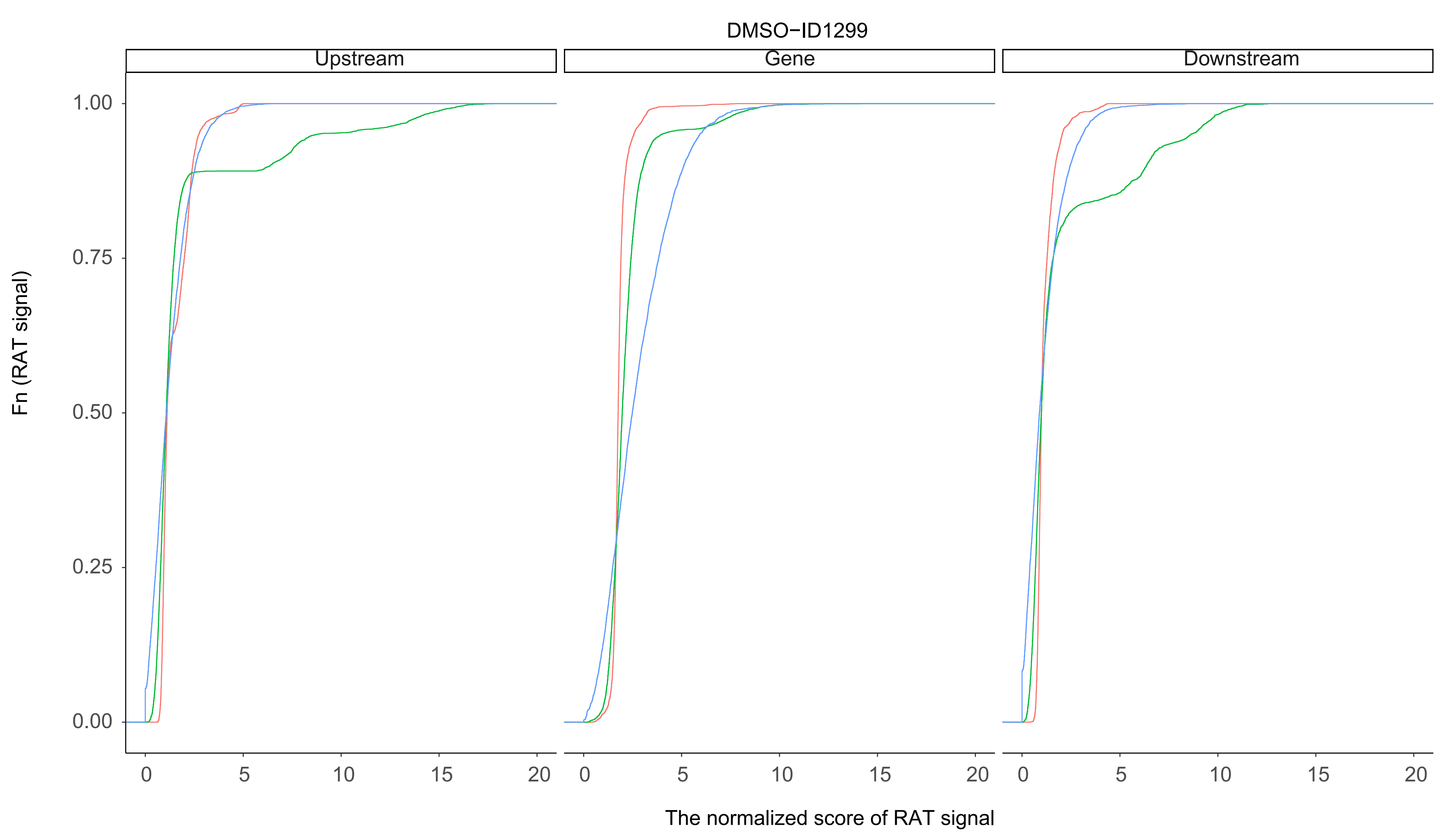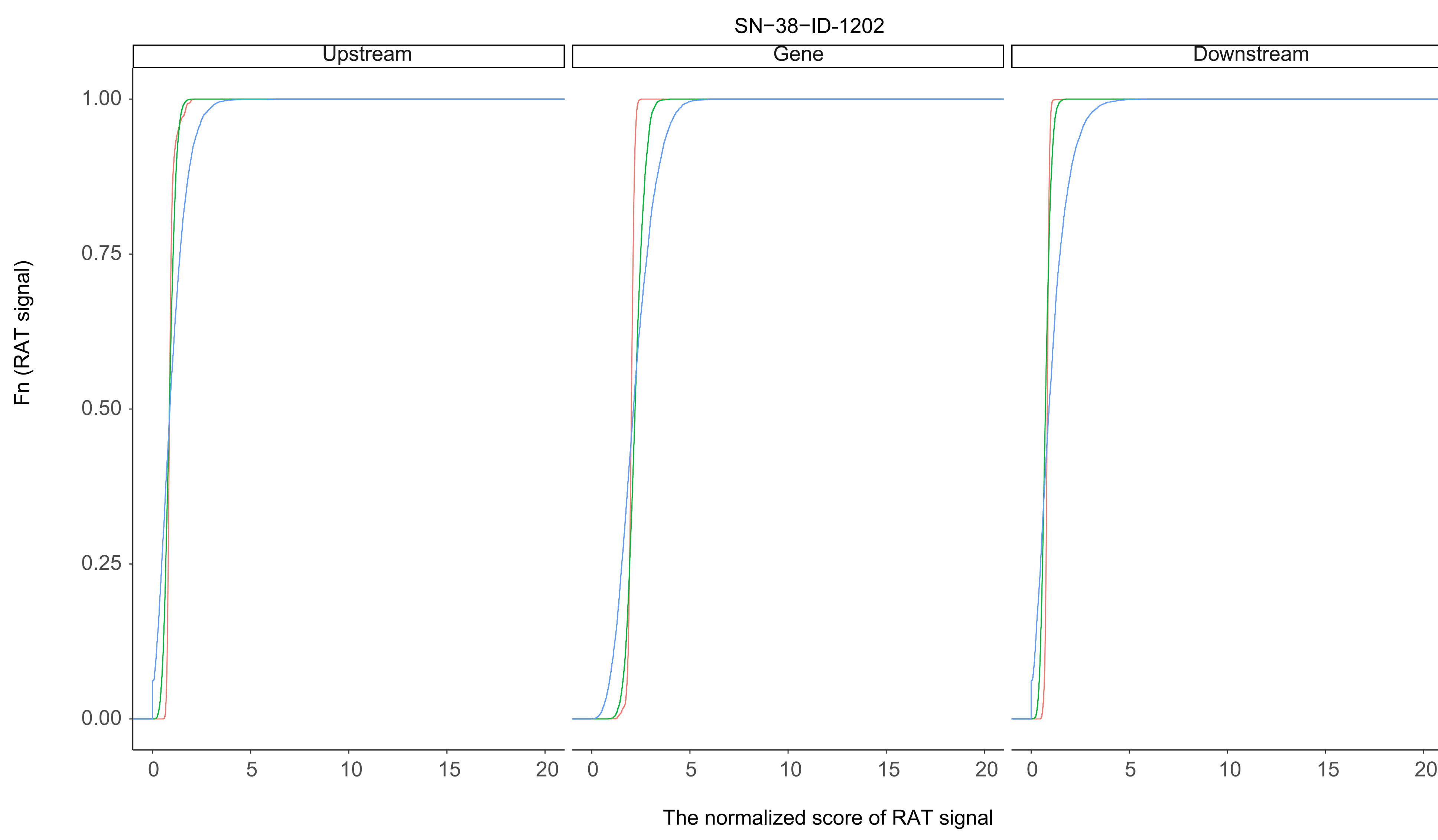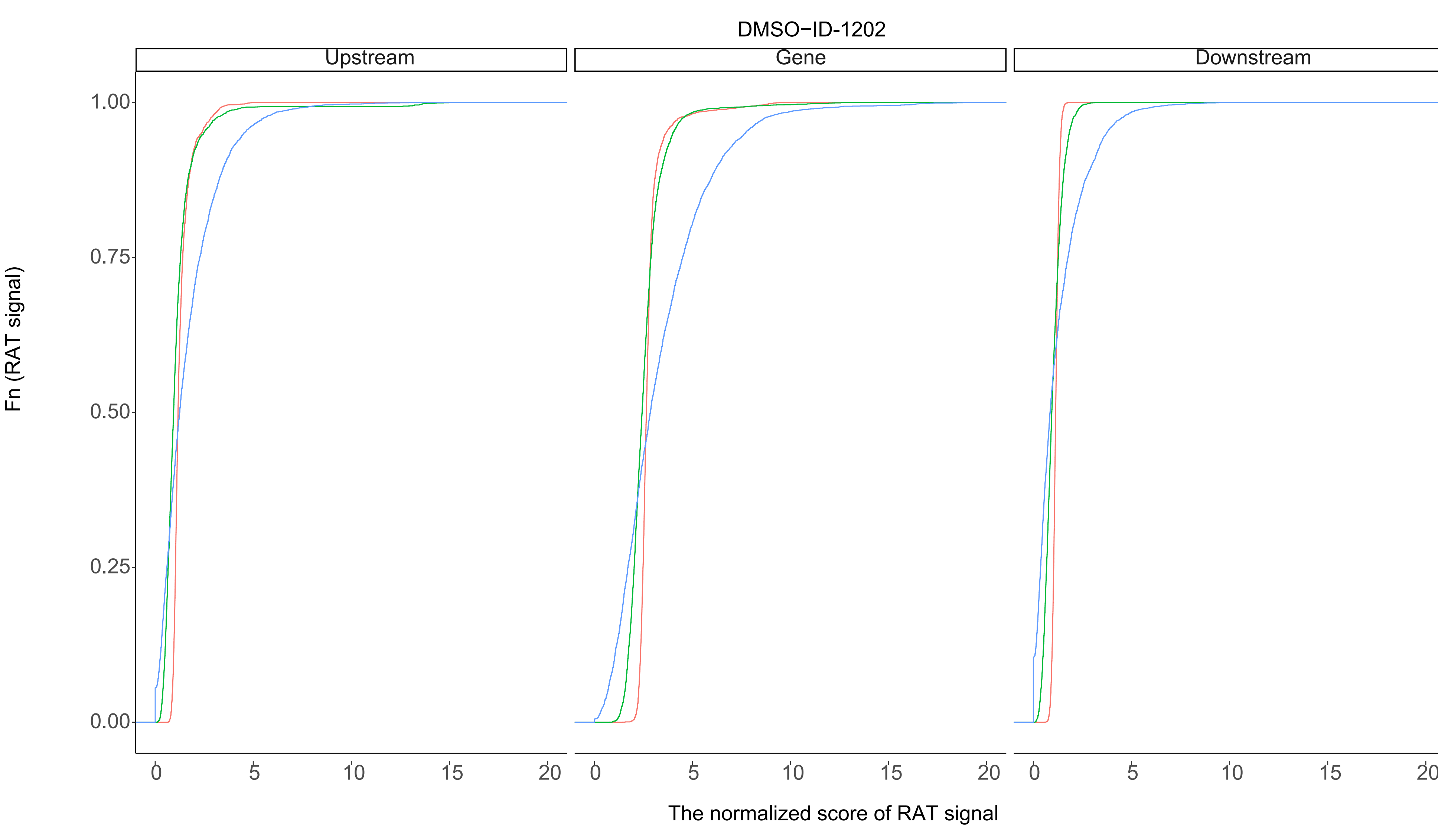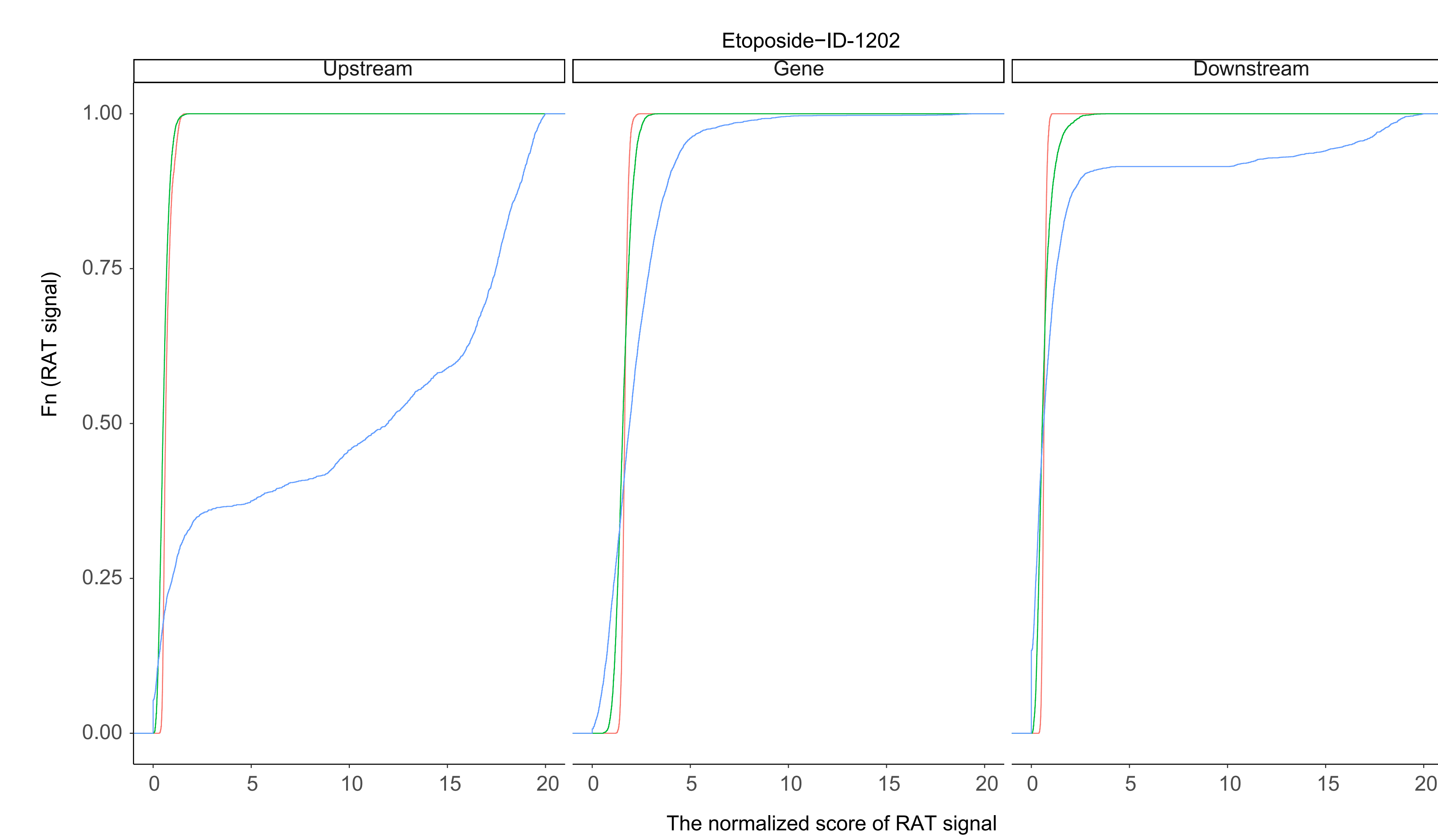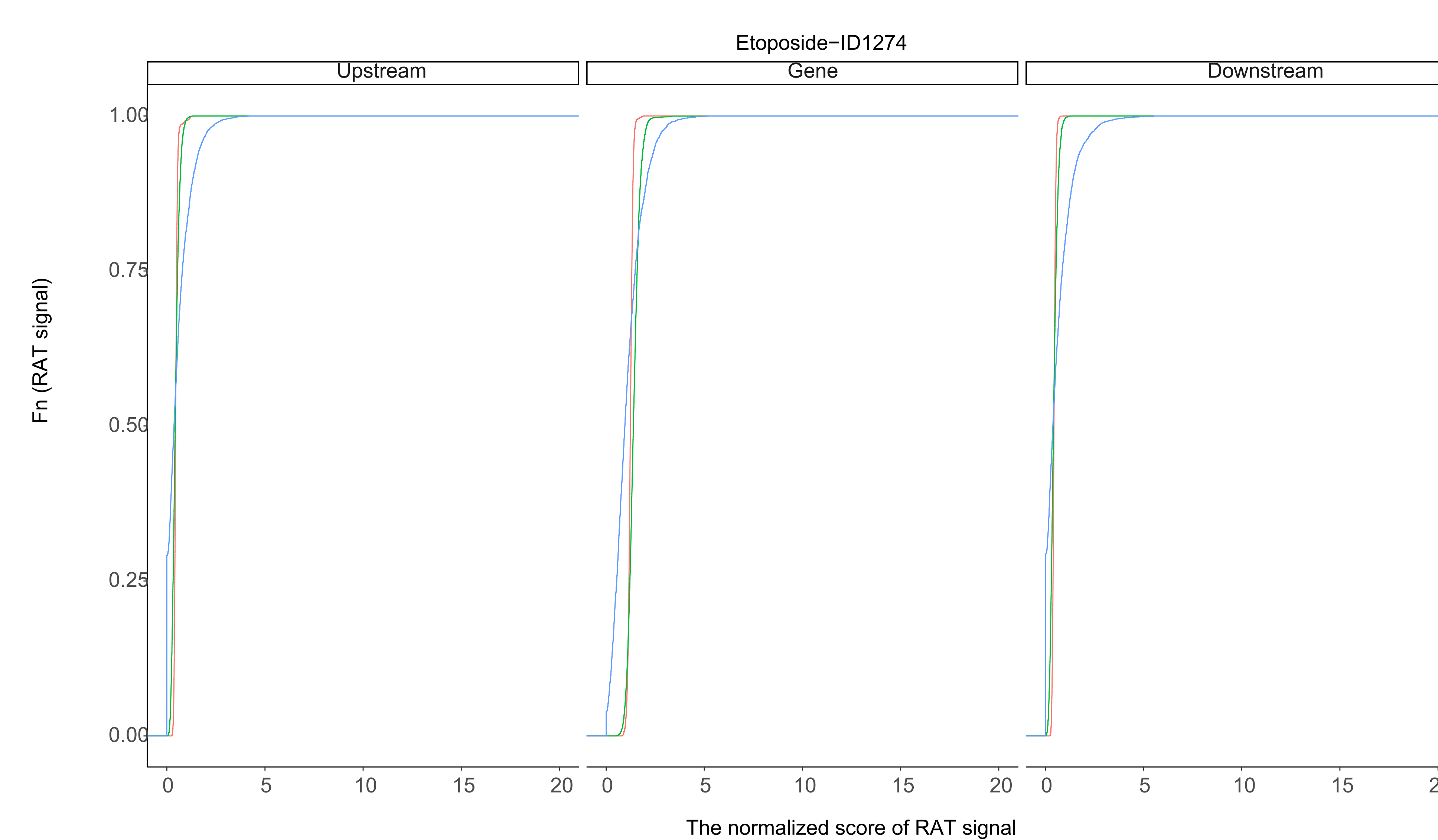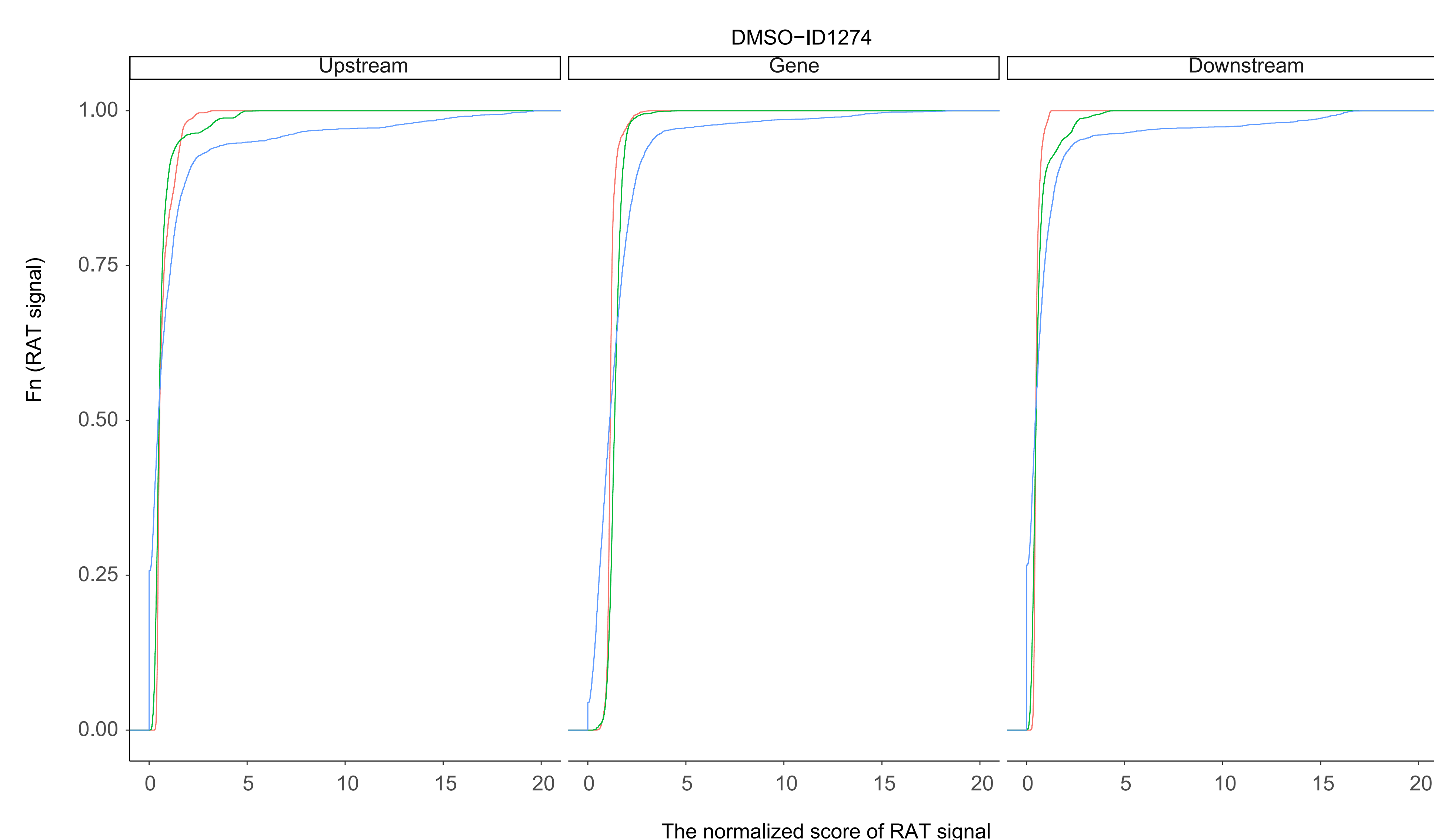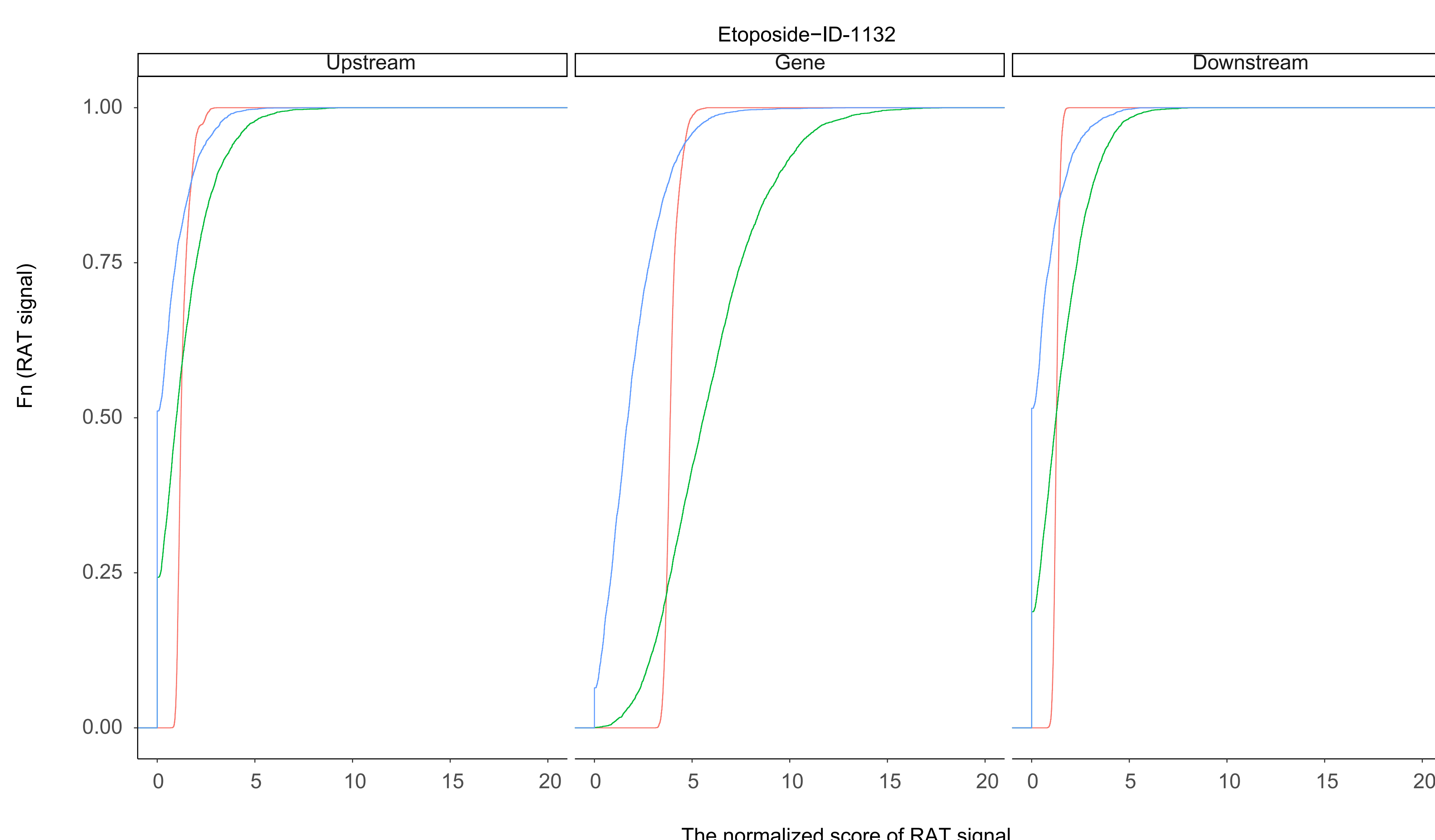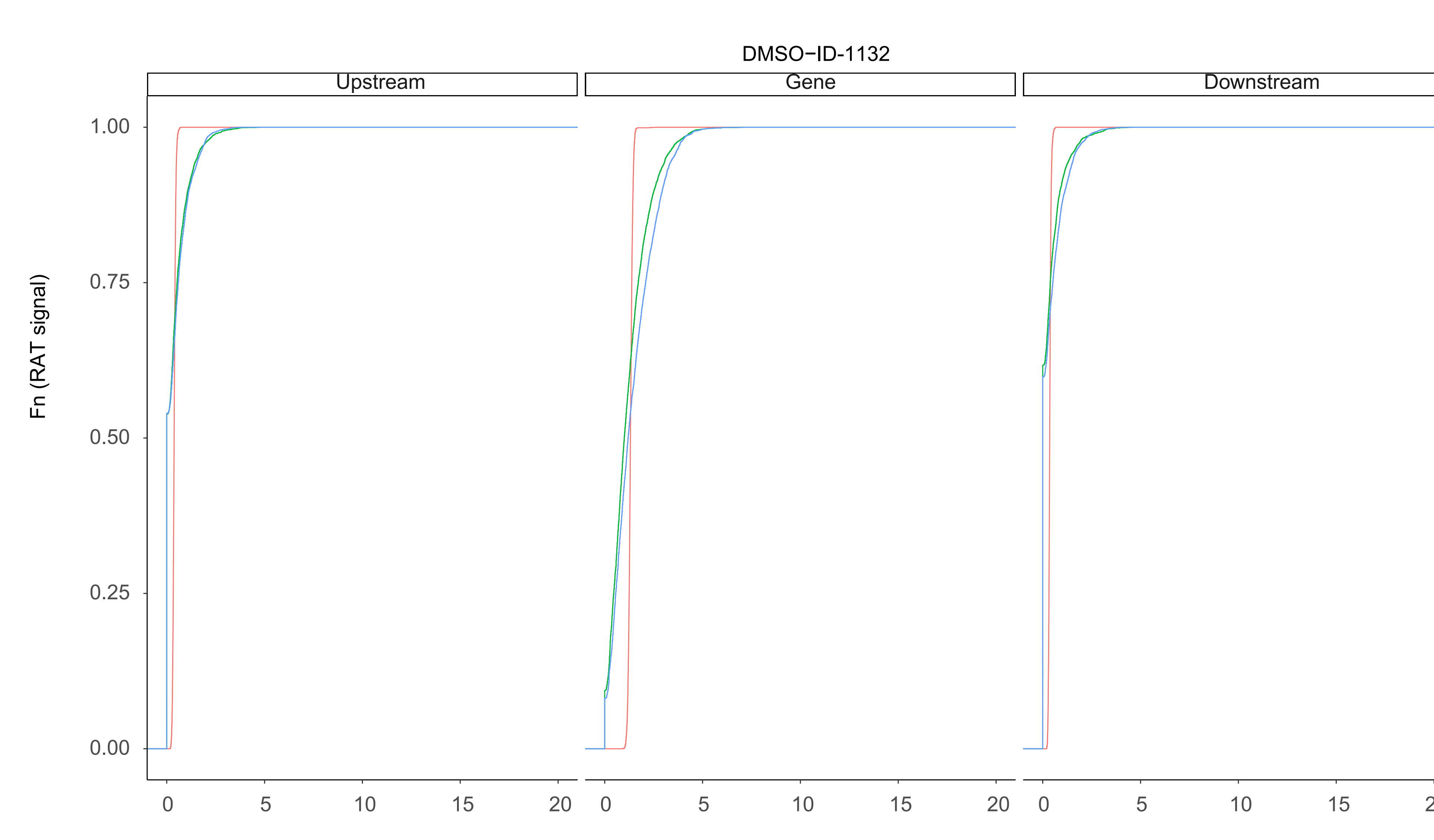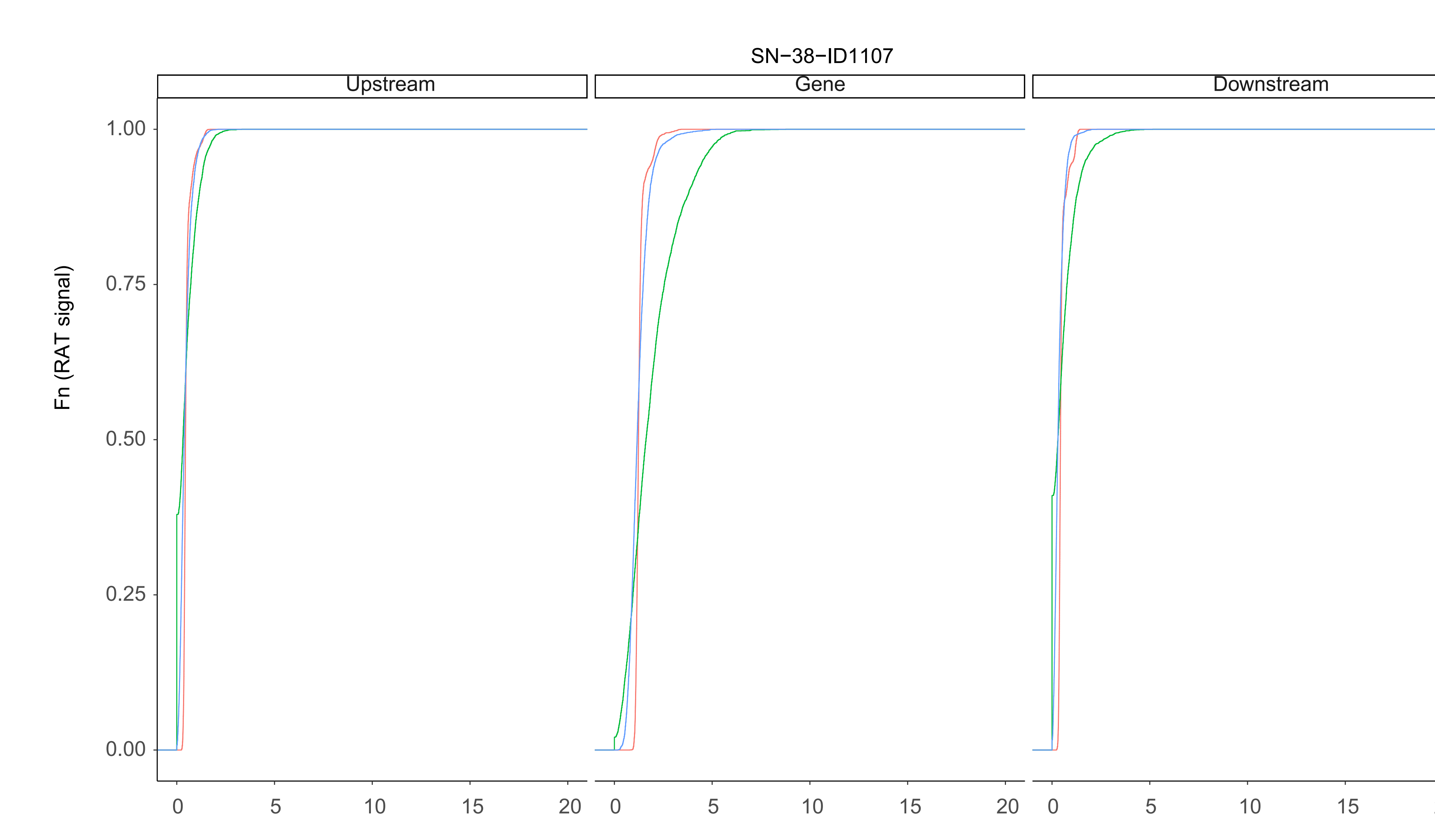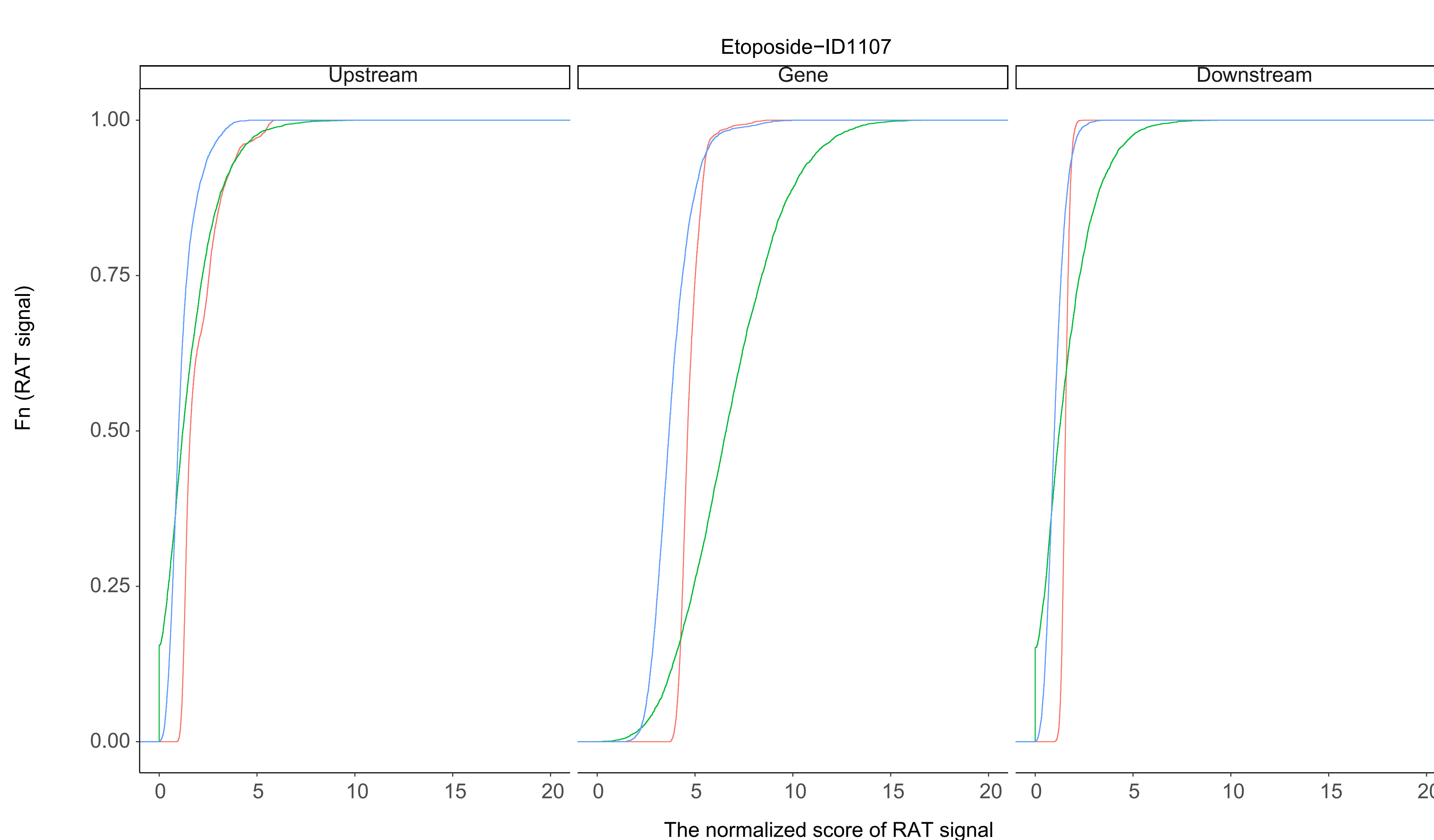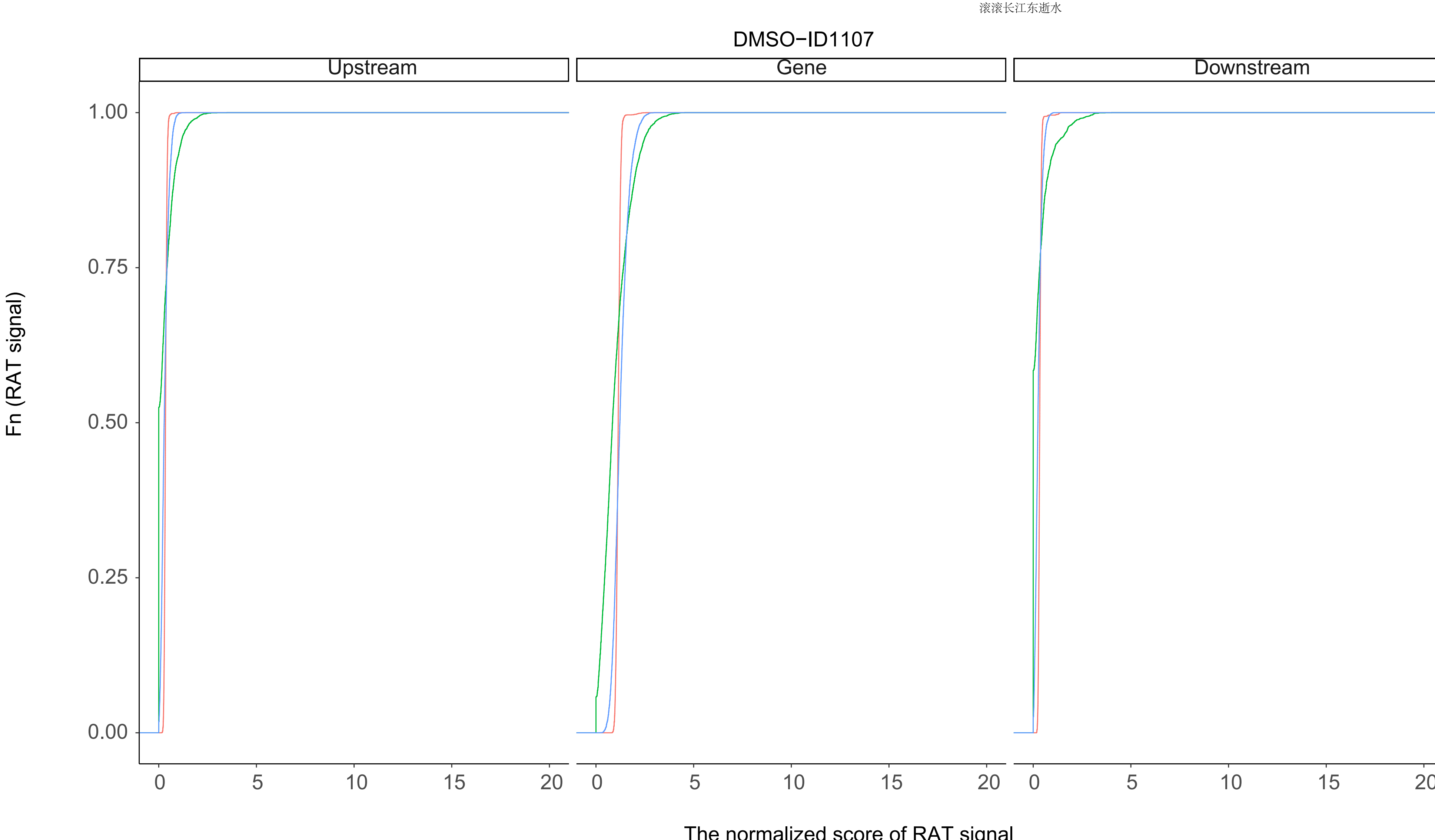

数据只供参考

Supplement: Supplementary file 4 — Additional file 4: Supplemental Figure S3. ECDF plots of ANARS in the boundaries and upstream or downstream 5kb flanking regions of positively and negatively co-expressed genes and background genes for each indicated vlincRNA-treatment combination. [file 12915_2021_1044_MOESM4_ESM.pdf]

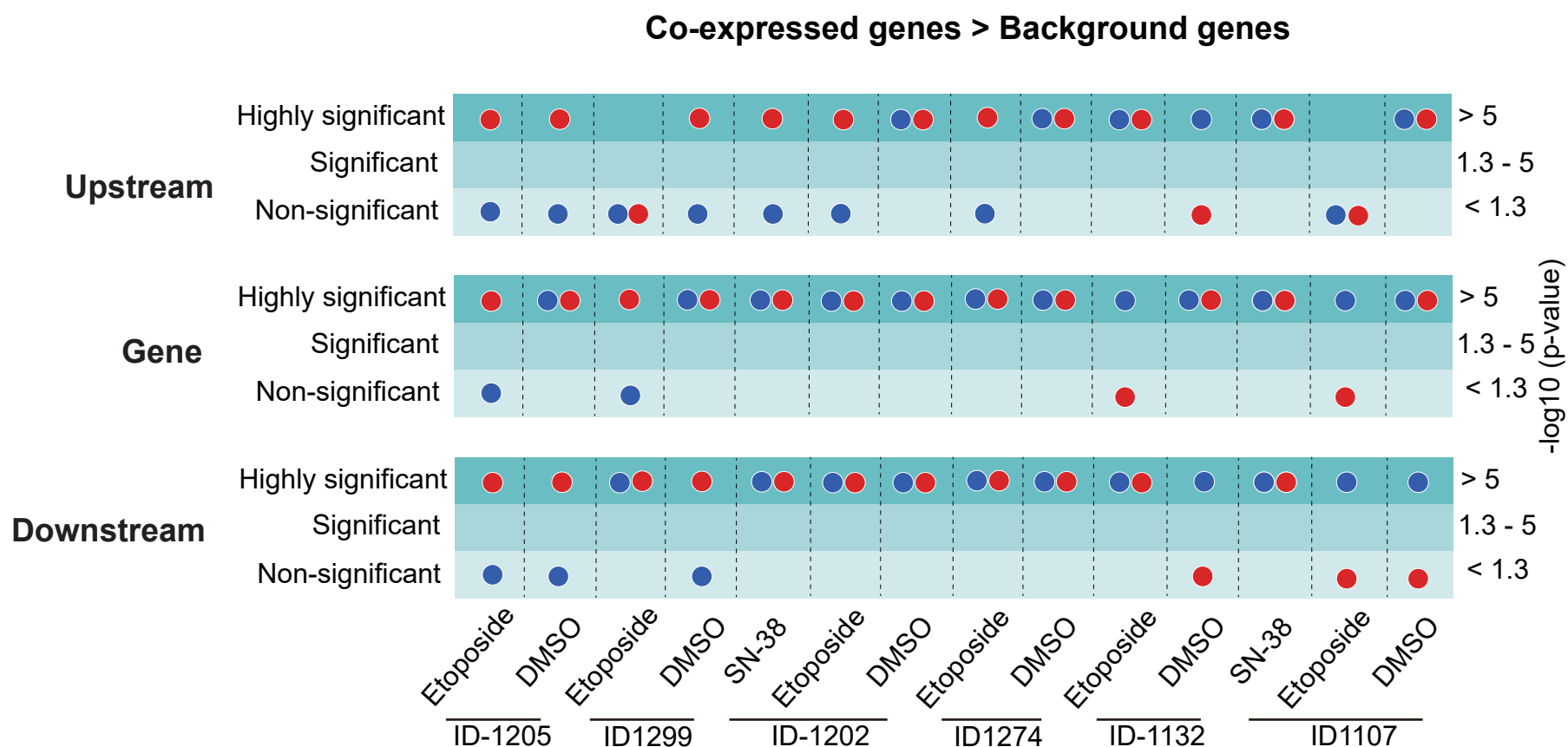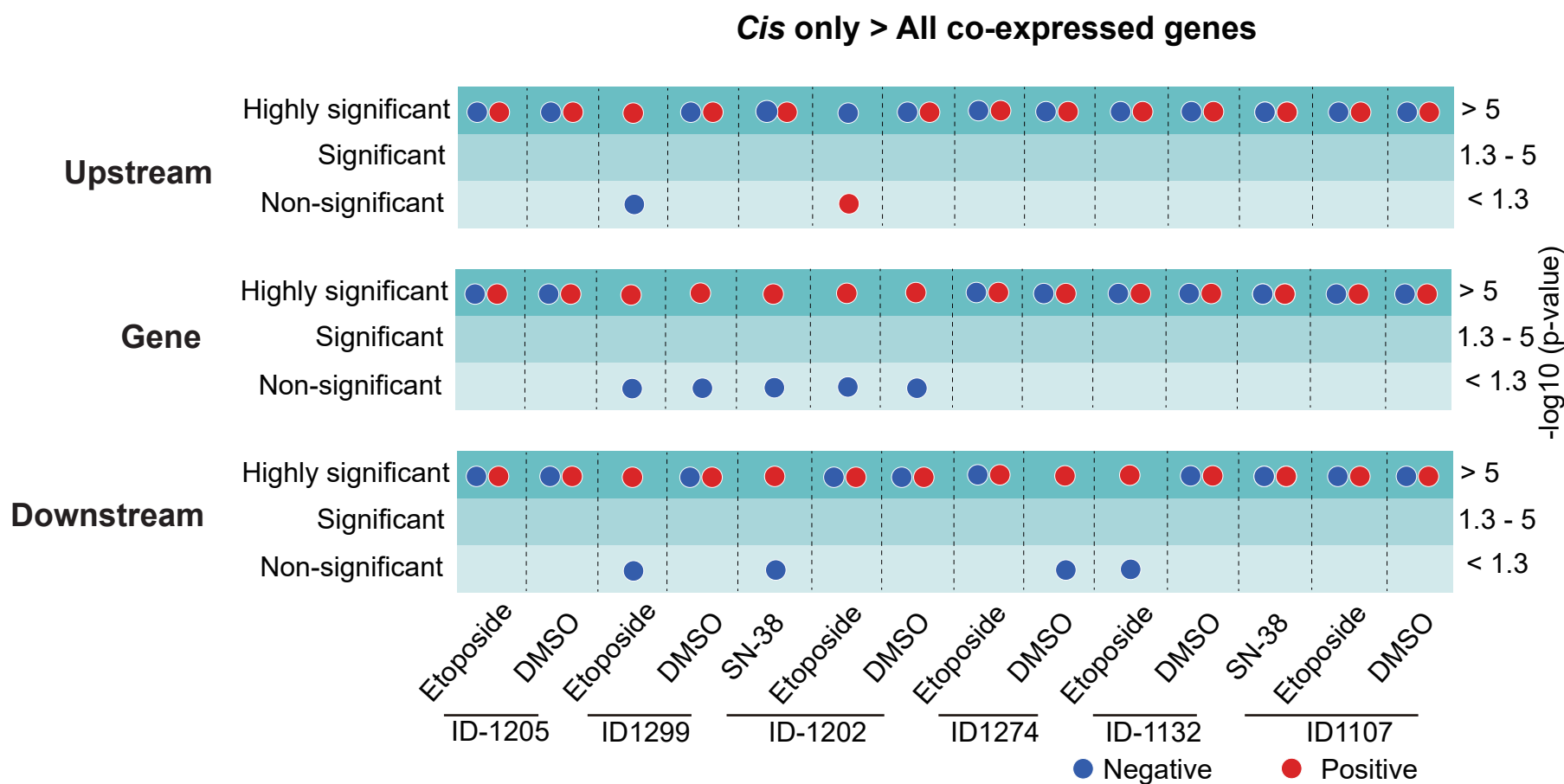

Supplement: Supplementary file 5 — Additional file 5: Supplemental Figure S4. Distribution of the statistical significance of enrichment of ANARS in the co-expressed vs the background genes (top) and cis only vs all co-expressed genes (bottom) for gene boundaries and upstream or downstream 5kb flanking regions among all indicated vlincRNA-treatment combinations. The p values were calculated based on the top 30% of the ranked ANARS values in each treatment as described in text and Methods. [file 12915_2021_1044_MOESM5_ESM.pdf]
