# Supplementary material for: Very long intergenic non-coding (vlinc) RNAs directly regulate multiple genes in cis and trans
Source: BMC Biol. 2021 May 20;19:108. doi: 10.1186/s12915-021-01044-x (PMC8139166; doi:10.1186/s12915-021-01044-x)
Supplement: Supplementary file 3 — Additional file 3: Supplemental Figure S2. A flow chart diagram of calculating average normalized aggregated RAT score (ANARS) for gene boundaries and upstream or downstream 5kb flanking regions. [file 12915_2021_1044_MOESM3_ESM.pdf]

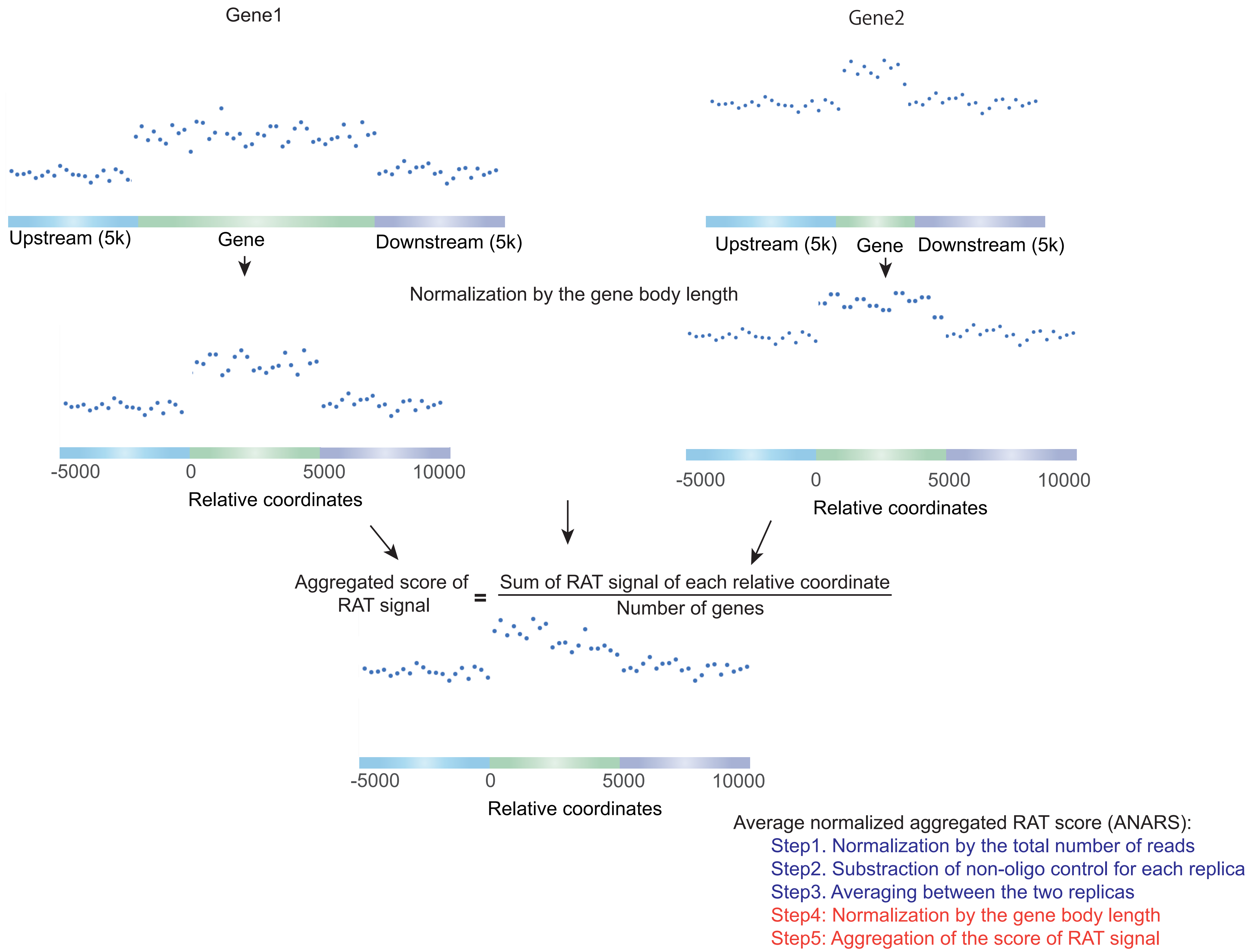

Gene1
Gene2
Upstream (5k)
Gene
Downstream (5k)
Upstream (5k)
Gene
Downstream (5k)
Normalization by the gene body length
-5000
0
5000
10000
Relative coordinates
-5000
0
5000
10000
Relative coordinates
Aggregated score of RAT signal = Sum of RAT signal of each relative coordinate / Number of genes
-5000
0
5000
10000
Relative coordinates
Average normalized aggregated RAT score (ANARS):
Step1. Normalization by the total number of reads
Step2. Substraction of non-oligo control for each replica
Step3. Averaging between the two replicas
Step4: Normalization by the gene body length
Step5: Aggregation of the score of RAT signal
