# Supplementary material for: Very long intergenic non-coding (vlinc) RNAs directly regulate multiple genes in cis and trans
Source: BMC Biol. 2021 May 20;19:108. doi: 10.1186/s12915-021-01044-x (PMC8139166; doi:10.1186/s12915-021-01044-x)
Supplement: Supplementary file 6 — Additional file 6: Supplemental Figure S5. Boxplots of non-zero ANARS in the cis and all co-expressed genes for gene boundaries and upstream or downstream 5kb flanking regions and for all indicated vlincRNA-treatment combinations. [file 12915_2021_1044_MOESM6_ESM.pdf]

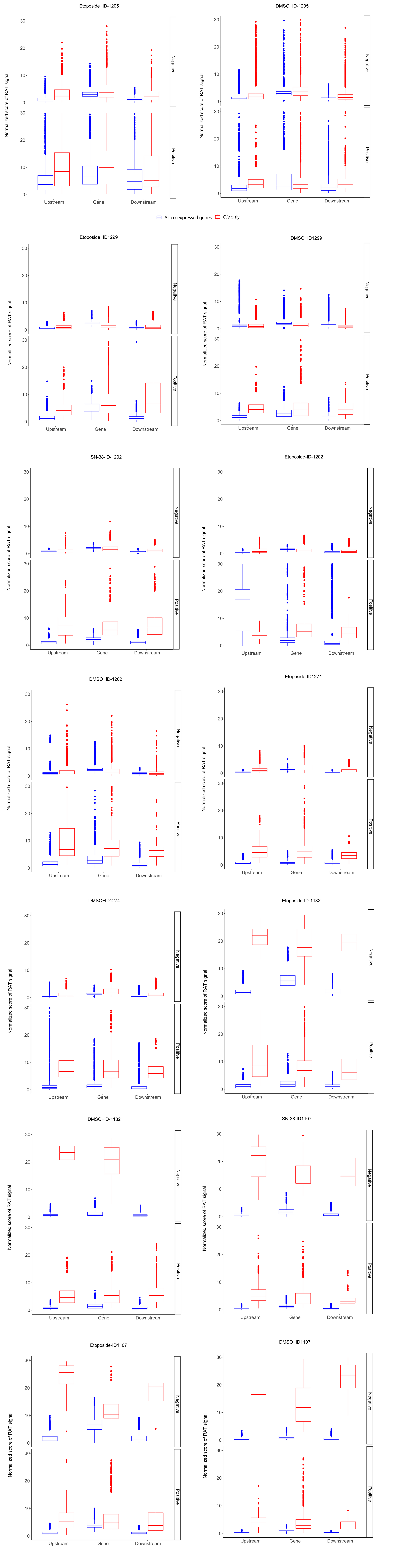
Etoposide-ID-1205
DMSO-ID-1205
All co-expressed genes
Cis only
Etoposide-ID1299
DMSO-ID1299
SN-38-ID-1202
Etoposide-ID-1202
DMSO-ID-1202
Etoposide-ID1274
DMSO-ID1274
Etoposide-ID-1132
DMSO-ID-1132
SN-38-ID1107
Etoposide-ID1107
DMSO-ID1107
Normalized score of RAT signal
Negative
Positive
Upstream
Gene
Downstream
